# Effects of Polyphenols on Oxidative Stress-Mediated Injury in Cardiomyocytes

**DOI:** 10.3390/nu9050523

**Published:** 2017-05-20

**Authors:** Rosanna Mattera, Monica Benvenuto, Maria Gabriella Giganti, Ilaria Tresoldi, Francesca Romana Pluchinotta, Sonia Bergante, Guido Tettamanti, Laura Masuelli, Vittorio Manzari, Andrea Modesti, Roberto Bei

**Affiliations:** 1 Department of Clinical Sciences and Translational Medicine, University of Rome “Tor Vergata”, 00133 Rome, Italy; rosannamatter@gmail.com (R.M.); monicab4@hotmail.it (M.B.); giganti@med.uniroma2.it (M.G.G.); ilaria3soldi@hotmail.com (I.T.); manzari@uniroma2.it (V.M.); modesti@med.uniroma2.it (A.M.); 2 IRCCS “S. Donato” Hospital, San Donato Milanese, Piazza Edmondo Malan, 20097 Milan, Italy; FrancescaRomana.Pluchinotta@cardio.chboston.org (F.R.M.); sonia.bergante@unimi.it (S.B.); guido.tettamanti@grupposandonato.it (G.T.); 3 Department of Experimental Medicine, University of Rome “Sapienza”, 00164 Rome, Italy; laura.masuelli@uniroma1.it; 4 Center for Regenerative Medicine (CIMER), University of Rome “Tor Vergata”, 00133 Rome, Italy

**Keywords:** polyphenols, oxidative stress, cardiovascular disease, cardiomyocytes

## Abstract

Cardiovascular diseases are the main cause of mortality and morbidity in the world. Hypertension, ischemia/reperfusion, diabetes and anti-cancer drugs contribute to heart failure through oxidative and nitrosative stresses which cause cardiomyocytes nuclear and mitochondrial DNA damage, denaturation of intracellular proteins, lipid peroxidation and inflammation. Oxidative or nitrosative stress-mediated injury lead to cardiomyocytes apoptosis or necrosis. The reactive oxygen (ROS) and nitrogen species (RNS) concentration is dependent on their production and on the expression and activity of anti-oxidant enzymes. Polyphenols are a large group of natural compounds ubiquitously expressed in plants, and epidemiological studies have shown associations between a diet rich in polyphenols and the prevention of various ROS-mediated human diseases. Polyphenols reduce cardiomyocytes damage, necrosis, apoptosis, infarct size and improve cardiac function by decreasing oxidative stress-induced production of ROS or RNS. These effects are achieved by the ability of polyphenols to modulate the expression and activity of anti-oxidant enzymes and several signaling pathways involved in cells survival. This report reviews current knowledge on the potential anti-oxidative effects of polyphenols to control the cardiotoxicity induced by ROS and RNS stress.

## 1. Introduction

Cardiovascular diseases are the main cause of mortality and morbidity in the world [1]. Hypertension, ischemia, diabetes, anti-cancer drugs contribute to heart failure through oxidative and nitrosative stresses which induce nuclear and mitochondrial DNA damage, denaturation of intracellular proteins, lipid peroxidation and inflammation in cardiomyocytes [2,3]. Oxidative or nitrosative stress-mediated injury lead to cardiomyocytes apoptosis or necrosis [2,3]. Reactive oxygen (ROS) and nitrogen species (RNS) concentration is dependent on their production and on the expression and activity of anti-oxidant enzymes [2]. ROS production is influenced by many factors, such as dysfunction of oxidative enzymes (xanthine oxidase (XO), aldehyde oxidase, nicotinamide adenine dinucleotide phosphate (NADPH) oxidase and uncoupled nitric oxide synthase (NOS)), dysregulation of mitochondria, microsomes and/or nuclei transport, neutrophil activation, arachidonic acid metabolism and auto-oxidation of catecholamines, flavins, quinones and proteins [4,5]. The main ROS and RNS are superoxide, hydrogen peroxide, hydroxyl radical, nitric oxide (NO) and peroxynitrite. The production of hydrogen peroxide regulates the expression of genes, in particular those activated by nuclear factor-kappa B (NF-κB), and the overload of Ca^2+^ levels in cardiomyocytes subjected to heart failure. The nitric oxide initiates lipid peroxidation and produces, by interacting with superoxide anion, peroxynitrite which is implicated in atherosclerosis [4]. Peroxynitrites trigger lipid peroxidation, protein oxidation, nitration and activation of matrix metalloproteinases (MMPs) [6]. However, NO can also maintain inactive the oxidant enzymes (XO and NADPH oxidase) and balance the superoxide/NO ratio [6]. In addition, iron and copper are the principal metal ions that induce the production of ROS by Fenton or Haber-Weiss reactions. Iron and copper enhance the lipid peroxidation and production of free radicals, including alkyl, alkoxy, peroxy and hydroxyl radicals. Conversely, selenium is an important anti-oxidant ion which regulates the glutathione peroxidase (GSH-Px) [4].

The mitochondria are identified as a major source of ROS production in several organs with high metabolic activity, for example in the heart [7]. Enzymes that produce ROS, both in membrane (NADPH oxidase) and in matrix (tricarboxylic acid cycle, TCA) are present in the mitochondria. Under normal conditions, ROS levels are low for the intra-mitochondrial presence of anti-oxidant systems. The increase of ROS production simultaneously to the inhibition of anti-oxidant systems and/or of ETC (electron transport chain) complex increase ROS levels [7]. Furthermore, sirtuins (SIRTs), localized in the mitochondria, play an essential role in cardiovascular disease and influence energy metabolism, DNA repair and oxidative stress. Sirtuins induce the deacetylation of forkhead box O (FoxO), NF-κB, protein kinase B (Akt), p53, superoxide dismutase (SOD), and members of ETC complex I and influence fatty acid oxidation, cardiac hypertrophy, ischemia/reperfusion (I/R) injury, apoptosis, oxidative stress and autophagy in cardiomyocytes [8]. Angiotensin (Ang) II, platelet-derived growth factor (PDGF), and tumor necrosis factor (TNF)-α induce the production of ROS in the cardiac muscle through the NADPH oxidase and then cardiomyocytes apoptosis, cardiac hypertrophy, reduction of myofilament sensitivity and cardiac contractility [9].

Several enzymatic and non-enzymatic mechanisms balance the production of ROS and transform ROS into non-toxic molecules in myocardium, as in other organs [9]. An anti-oxidant enzyme is SOD, which is present in three forms, copper-zinc SOD (CuZnSOD or SOD1), manganese SOD (MnSOD or SOD2) and extracellular SOD (EC-SOD or SOD3) [8]. Other enzymes, including the catalase (CAT) and GSH-Px reduce the production of ROS. SOD converts superoxide in hydrogen peroxide, which is transformed into water and oxygen by CAT and GSH-Px. Thioredoxin and thioredoxin reductase form an additional system that blocks ROS production through protein-disulfide oxidoreductase activity. Intracellular non-enzymatic anti-oxidants are vitamins E and C, β-carotene, ubiquinone, lipoic acid, urate and glutathione [6,10]. Anti-oxidant enzymes are activated during oxidative stress and after activation of cytokines. However, these enzymes are down-regulated during the end stage of heart failure [10].

**Figure 1:**
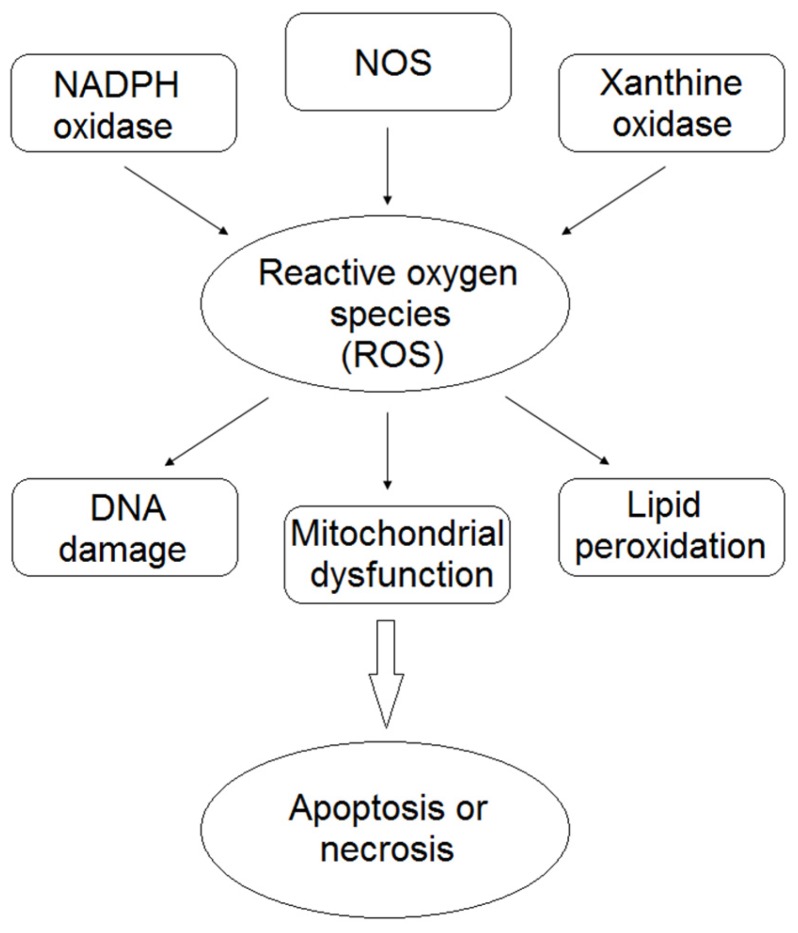
Generation of reactive oxygen species (ROS) and their cellular effects.

Recently, many studies identified the potential anti-oxidant effects of polyphenols in cardiac diseases. Polyphenols can reduce the cardiac damage due to ROS and RNS production [11] (Figure 1). In this review, we report the last ten years researches on the potential effects of polyphenols in modulating the cardiotoxicity induced by ROS or RNS.

## 2. Polyphenols

### 2.1. Classification

Polyphenols are a large group of natural compounds found in foods and beverages of plant origin (fruits, vegetables, cereals, herbs, spices, legumes, nuts, olives, chocolate, tea, coffee, and wine) [12,13].

**Figure 2:**
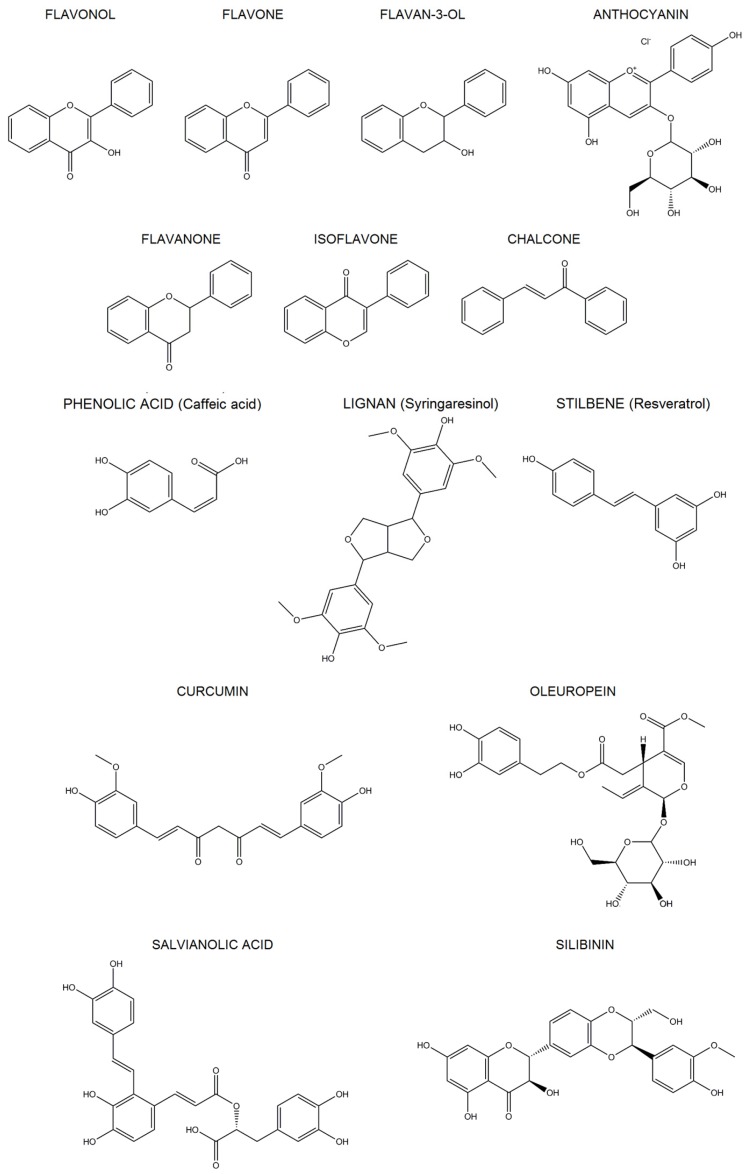
Chemical structure of polyphenols.

Polyphenols are classified in flavonoids and non-flavonoids, according to the number of phenol rings and structural elements bound to these rings. The most important classes of flavonoids found in foods are flavonols, flavones, flavan-3-ols, anthocyanins, flavanones and isoflavones. The subclasses of dihydroflavonols, flavan-3,4-diols, chalcones, dihydrochalcones, and aurones are minor components of our diet. The most important classes of non-flavonoids are phenolic acids, stilbenes and lignans [14,15,16] (Figure 2).

### 2.2. Polyphenols and Oxidative Stress and Epigenetic Regulation

Epidemiological studies have shown associations between a diet rich in polyphenols and the prevention of human diseases [12,17,18,19,20,21]. Polyphenols are natural anti-oxidants present in the human diet. This activity is related to their metal ions chelating and free radical scavenger properties. Structural features of polyphenols allow them to act as direct free radical scavengers, such as the catechol group on the B-ring, the presence of hydroxyl groups at the 3 and 5 position and the 2,3-double bond in conjugation with a 4-oxofunction of a carbonyl group in the C-ring [22]. However, polyphenols can also behave as pro-oxidants at high doses or in the presence of metal ions, leading to DNA degradation. However, there is no evidence of sistemic pro-oxidant effect of polyphenols in humans [23].

It is of note that the direct anti-oxidant activity of polyphenols appears to be ineffective in vivo, because of the low bioavailability and kinetic constraints, and it has been proposed that the beneficial effects observed are due to an indirect anti-oxidant effect rather than to their direct free radical scavenger properties. Indeed, it has been demonstrated that polyphenols possess an indirect anti-oxidant capacity by modulating genes expression and by inducing the endogenous anti-oxidant enzymatic defense system [24,25,26]. The indirect modality of oxidative stress protection and the health benefits exerted by polyphenols are due to the hormetic mechanism of action. Polyphenols activate adaptive cellular response pathways (hormetic pathways) that induce the expression of genes encoding for anti-oxidant enzymes, phase-2 enzymes, protein chaperones and survival-promoting proteins. Example of these pathways are the Keap1/Nrf2/ARE, the Sirtuin-FoxO and the NF-κB pathways [27,28]. In particular, it has been demonstrated that polyphenols activate the Keap1/Nrf2/ARE pathway through an oxidative mechanism. In fact, polyphenols must be metabolized into electrophilic compounds for inactivating the inhibitor Keap 1 and thus activating Nrf2, a transcription factor regulating the expression of most Phase II and some Phase III genes [26]. By transcription-mediated signalling, polyphenols exert long-lasting effects as compared to other direct anti-oxidant agents [27,29]. However, the hypothesis of activation of Phase II enzymes by polyphenols has not been proven in human yet [28,30].

Several studies reported that epigenetic mechanisms are involved in the development and progression of different diseases, including cardiovascular diseases [31]. Polyphenols can reduce the risk of cardiovascular disease by interacting with signaling cascades and epigenetic factors [32]. Several evidence reported that epigenetic mechanisms are involved in hormesis-like responses [33]. Epigenetic modifications depend on the cell type and the stage of the cardiovascular disease. For example, the global DNA is hypermethylated in the early stage of atherosclerosis and hypomethylated in the atherosclerotic lesions, while the peripheral blood leucocytes show global hypermethylation of DNA. In addition, the methylation or acetylation of DNA regulatory regions up- or down-regulate the expression of genes involved in cardiovascular diseases [34]. Polyphenols can reduce DNA methylation, as well as histone modifications and can modify the expression of transcription factors by epigenetic regulation [32,34,35,36]. Polyphenols silence chromatin by the inhibition of DNA methyltransferases and the activation of class III histone deacetylases (commonly called sirtuins) in vascular endothelium. These effects occur in atherosclerosis and other cardiovascular diseases [36,37].

MicroRNAs (miRNAs) are non-coding RNAs which modify the transcription with no interference with DNA sequences [34]. miRNAs regulate gene transcription through the degradation or repression of mRNA. Various miRNAs are also associated with cardiovascular diseases as biomarkers [34]. For example, increased levels of miRNA-1, miRNA-133b and miRNA-499 are observed in acute myocardial infarction [38,39]. Elevated levels of miRNA-217 are associated with inhibited expression of SIRT1 in atherosclerosis [40]. However, the levels of several miRNAs can also decrease in cardiovascular diseases. In fact, miRNA-126 and miRNA-145 levels decrease in patients with coronary artery disease [41]. Polyphenols modulate the expression of several miRNAs [42]. For example, resveratrol regulates the cardiac function through the expression of miRNA-20b, miRNA-149, miRNA-133, miRNA-21, and miRNA-27 [32]. In addition, it has been reported that resveratrol exerts cardioprotective effects through the up-regulation of miRNA-21 and SIRT1 expression in ischemic rats. miRNA-21 regulates cardiac remodeling, cardiac cell growth and apoptosis [43].

In vitro studies underlying molecular mechanisms through which polyphenols affect oxidative stress-induced cardiotoxicity are reported below.

## 3. In Vitro Effects of Polyphenols Against Oxidative Stress-Induced Cardiotoxicity

### 3.1. Flavonoids

#### 3.1.1. Flavonols

Flavonols are the most common flavonoids present in plant-derived foods, wine and tea. Quercetin, kaempferol and myricetin are the main flavonols [17].

Several studies investigated the effect of flavonols against H_2_O_2_-induced cardiotoxicity. Quercetin or 3′-*O*-methyl quercetin treatment (30 µM) or rhamnetin, from berries of *Rhamnus petiolaris*, (1, 3, 5 µM) treatment of rat/H9c2 cardiomyocytes exposed to H_2_O_2_ decreased ROS production and number of death cells by up-regulating the NQO-1, HO-1, TR (thioredoxin reductase 1), and total glutathione *S*-transferase (GST) activities or by inhibiting MAPKs activation [44,45,46]. Conversely, 3′,4′-dihydroxyflavonol (DiOHF, 10 µM) protected rat cardiomyocytes against H_2_O_2_-induced oxidative stress by increasing the phosphorylation of MAPKs and Akt and regulated the activation of the reperfusion injury survival kinase (RISK) pathways [47].

Doxorubicin (DOX)/Adriamycin is an anthracycline antibiotic used in chemotherapy and it is associated with cardiotoxicity. The treatment of H9c2 cardiomyocytes with quercetin (50, 100 µM), isorhamnetin (6.25–25 µg/mL), kaempferol (20 μM) or dihydromyricetin (50 µM, a flavonoid extracted from *Ampelopsis grossedentata*), decreased the DOX-induced apoptosis, ROS production, lipid peroxidation and NADPH oxidase activity. Isorhamnetin also increased the production of anti-oxidant enzymes and modulated MAPK activity [48,49,50,51].

Quercetin pretreatment (20 µM) of H9c2 cardiomyocytes significantly attenuated trauma-induced apoptosis, TNF-α levels, ROS production and Ca^2+^ levels [52].

PKC_Ɛ_, a member of the PKC family of serine and threonine kinases, regulates several processes, including mitogenesis, cell survival, metastasis and transcriptional regulation. The treatment of rat cardiomyocytes with quercetin (40 µM) or kaempferol (20 μM) before anoxia/reoxygenation (A/R) mediated-injury increased PKC_Ɛ_, cells viability and the expression of SIRT1, decreased apoptosis and ROS generation and restored the mitochondrial membrane potential [53,54]. The incubation of sheep myocardial tissues with DiOHF (10^−4^–10^−6^ M) blocked ROS production after myocardial I/R injury [55].

Taxifolin (5–50 µM), a derivative of dihydroquercetin, reduced hypertrophy, ROS generation and protein synthesis induced by Ang II in neonatal rat cardiomyocytes [56].

The effects of quercetin and hydroxytyrosol, present in the leaves of olive (*Olea europaea* L.), have been evaluated in H9c2 cardiomyocytes exposed to xanthine/XO-induced oxidative stress. Both quercetin and hydroxytyrosol (0.1, 10 µg/mL) reduced the number of death cells, ROS generation and the phosphorylation of the MAPK-activated protein kinase 2 (MAPKAPK-2) after exposure to xanthine/XO [57].

#### 3.1.2. Flavones

Flavones are found in plants as 7-*O*-glycosides. The most abundant flavones in foods are apigenin (parsley, celery, onion, garlic, pepper, chamomile tea) and luteolin (Thai chili, onion leaves, celery). Others flavones, less abundant in edible plants, are tangeretin, nobiletin, baicalein, wogonin and chrysin [17].

The effect of apigenin and vitexin (a flavone glycoside of apigenin isolated from the leaf of *Crataegus pinnatifida Bunge*) on A/R injury was recently assessed in H9c2 and neonatal rat cardiomyocytes. Apigenin (40 µM) or vitexin (10, 30 and 100 µM) were able to increase cells viability and to decrease ROS generation, cells apoptosis and necrosis [58,59].

Vitexin has also been shown to possess significant protective effects against myocardial I/R injury in isolated rat hearts. Hearts were perfused with vitexin (50, 100, 200 µM) for 20 min before ischemia. Treatment with vitexin inhibited I/R-induced reduction of coronary flow and decreased I/R-induced histopathological alterations of myocardium, inflammatory cytokines and it inhibited apoptosis [60]. Baicalein from the root of *Scutellaria baicalensis* was analyzed for its anti-oxidative effects in chick cardiomyocyte models during hypoxia, simulated I/R, or mitochondrial complex III inhibition with antimycin A. Baicalein (10–100 µM) was able to scavenge ROS and to decrease the number of death cells in all different models [61]. Recently, it has been proven that baicalein protected H9c2 cardiomyocytes and also human embryonic stem cells-derived cardiomyocites (hESC-CMs) against oxidative stress-induced cell injury. Baicalein (10, 30 µM) decreased ROS generation and the number of death cells and activated Nrf2 pathway [62].

Conversely, Chang et al. reported that preconditioning with baicalein (10 µM) for a brief time before I/R injury protected chick embryonic ventricular myocytes from apoptosis through mitochondrial pro-oxidant mechanisms. Thus, the authors hypothesized that the oxidant-mediated protective effects of baicalein was probably due to the modulation of mitochondrial pro-oxidant signaling as well [63]. Baicalein (1 µM) also reduced H/R-induced myocardium necrosis and apoptosis through μ- and δ-opioid but not κ-opioid receptors pathways in chick embryonic cardiomyocites [64].

#### 3.1.3. Flavan-3-Ols

The flavan-3-ol subclass includes several compounds with different chemical structures that can be divided in monomers, (+)-catechin, (−)-epicatechin, (+)-gallocatechin, (−)-epigallocatechin, (−)-epicatechin-3-*O*-gallate, (−)-epigallocatechin-3-*O*-gallate (EGCG), and polymers (proanthocyanidins). They are found in fruits, berries, cereals, nuts, chocolate, red wine, and tea [17].

Several studies investigated the effects of flavan-3-ols in cardiomyocytes exposed to exogenous and endogenous oxidant stress. Pretreatment of rat cardiomyocytes exposed to H_2_O_2_ with EGCG (20, 50 µM or 50–100 mg/L) and theaflavin-3,3′ digallate (TF3, 20 µM) decreased ROS production, cellular damage and apoptosis through the inhibition of telomere dependent apoptotic pathway [65,66,67,68]. EGCG (50 µM) was also able to protect H9c2 cardiomyocytes from H_2_O_2_-induced changes in the expression of β-catenin, *N*-cadherin and the gap junction protein Cx43 and to activate caveolin-1 by modulating Akt/GSK-3β signaling [66].

Other studies investigated the cardioprotective role of flavan-3-ols in cardiomyocytes exposed to DOX. EGCG (25, 38, 50, or 100 μM) and grape seed proanthocyanidin extract (GSPE, 50 μg/mL) protected rat cardiomyocytes against DOX-induced oxidative stress by reducing apoptosis, ROS generation and the distruption of mitochondrial membrane potential, and by increasing anti-oxidant enzymes and by reversing DOX-induced intracellular Ca^2+^ depletion in the sarcoplasmic reticulum [69,70,71]. GSPE prevented cardiotoxicity induced by DOX, without interfering with DOX anti-neoplastic activity in primary cultures of cardiomyocytes [71]. Two studies showed the role of flavan-3-ols against toxicity induced by I/R injury. EGCG (10, 100 µM), and GSPE (50 μg/mL) protected rat and chick cardiomyocytes from I/R-induced apoptosis and cellular damage [72,73]. Similarly, Hirai et al. evaluated the cardioprotective effects of EGCG and the catechin epimer (GCG) in isolated guinea-pig Langendorff hearts subjected to I/R. EGCG (3 × 10^−5^ M) or GCG (3 × 10^−6^ M) was introduced into the perfusate 4 min before ischemia and during reperfusion. EGCG and GCG improved the recovery of left ventricular end diastolic pressure (LVDP), increased the tissue levels of ATP and phosphocreatine and inhibited apoptosis [74]. Wei and Meng analyzed the effect of EGCG against bisulfite/sulfite, an agent that induces sulfur- and oxygen-based free radicals and affects voltage-gated sodium (Na^+^) channels (VGSC), or lead in rat ventricular myocytes. EGCG (30 µg/mL) increased anti-oxidant enzymes and decreased ROS levels and lipid peroxidation in ventricular myocytes of rats exposed to sulfite or lead [75,76]. EGCG also protected Na^+^ channels against the oxidative damage induced by sulfite [75].

An in vitro study observed the effects of flavonoids isolated from *Lindera erythrocarpa* in H9c2 cardiomyocytes exposed to BSO (buthionine-(*S*,*R*)-sulfoximine), an inducer of oxidative stress and cell death. It was found that (−)-epicatechin (3 µM), avicularin (3 µM) and quercitrin (3 µM) had the ability to inhibit BSO-induced cardiomyocytes death [77].

#### 3.1.4. Anthocyanins

Anthocyanins are a subclass of more than 550 compounds. The most abundant anthocyanins are cyanidin, pelargonidin, delphinidin, peonidin, petunidin, and malvidin. They occur in two forms: the aglycone form (anthocyanidin) and the heteroside form (anthocyanin). They are found mainly in berries, cherries, red grapes, and currants [17].

Two studies evaluated the effects of malvidin, an anthocyanidine present in red wine, and cyanidin-3-*O*-glucoside (Cy3G) in isolated rat hearts after I/R. Pretreatment with malvidin (10^−7^ M) or Cy3G (20 μM) increased left ventricular (LV) pressure, reduced cells apoptosis and necrosis [78,79].

Louis et al. investigated the in vitro effect of blueberry phenol fractions (BF) to prevent the pathologic damage induced by norepinephrine (NE) in adult rat cardiomyocytes. NE induced cells hypertrophy and death by activating calpains. Pretreatment with BF (6.55 µg/mL) reduced activation of calpains and apoptosis and increased activity of anti-oxidant enzymes [80].

#### 3.1.5. Flavanones

Flavanones are found mainly in citrus fruits and occur as aglycones, mono- and di-glycosides. Naringenin and hesperetin are the most important aglycones; neohesperidin (hesperetin-7-*O*-neohesperidoside) and naringin (naringenin-7-*O*-neohesperidoside) are neohesperidosides, and hesperidin (hesperetin-7-*O*-rutinoside) and narirutin (naringenin-7-*O*-rutinoside) are rutinosides [17].

Naringenin (NGN) was proven to possess a cytoprotective effect against oxidative stress by up-regulating the transcription of Nrf2 and its target genes (GCL and NQO-1). Indeed NGN (50 µM) protected H9c2 cardiomyocytes from H_2_O_2_-mediated cells death, reduced lipid peroxidation and increased the activity of anti-oxidant enzymes [81].

Several studies demonstrated that naringin was able to protect H9c2 cardiomyocytes against high glucose (HG)-induced injury. Pretreatment with naringin (5 or 80 µM) increased cells viability and reduced the number of apoptotic cells, and dissipation of mitochondrial membrane potential (MMP). Naringin protected H9c2 cardiomyocytes by inhibiting ROS production and activation of MAPKs [82,83,84]. Naringin also confered protection against A/R-induced injury in H9c2 cardiomyocytes. Pretreatment with naringin (10, 20, 40 µg/mL) inhibited apoptosis and oxidative stress. Naringin protected H9c2 cardiomyocytes through the activation of ERK1/2, PKCδ, Akt and then the activation of Nrf2 signaling pathway [85].

Several studies analyzed the effect of naringenin-7-*O*-glucoside (NARG) extracted from Chinese traditional medicine herb *Dracocephalum Rupestre* against DOX-induced oxidative stress and apoptosis. NARG (5–40 µM) reduced DOX-induced apoptosis, necrosis, ROS generation and it up-regulated the expression of endogenous anti-oxidant enzymes in H9c2 cardiomyocytes [86,87,88].

Yang et al. demonstrated that hesperetin (25 µM) exerted an anti-apoptotic effect in LPS-stimulated H9c2 cardiomyocytes, through the mitochondria-dependent intrinsic apoptosis pathway [89].

#### 3.1.6. Isoflavones

Isoflavones are classified as phyto-estrogens for their chemical structure. They are found in soybeans, soy products, and leguminous plants. Isoflavones are present in plant food mainly as aglycones (genistein, daidzein) and glycosides (genistin, daidzin, puerarin) [17].

Puerarin, a bioactive daidzein glucoside derived from *Radix puerariniae*, was studied for its effects on Ang II-induced cardiac hypertrophy. In vitro pretreatment of neonatal murine cardiomyocytes with puerarin (50, 100 µM) decreased NADPH oxidase activity, inhibited ROS production and the activation of oxidative stress-related signaling pathways [90].

Calycosin, an O-methylated isoflavone derived from *Astragali radix*, has been evaluated for its effects on oxidative stress-induced apoptosis in H9c2 cardiomyocytes. It has been found that pretreatment with calycosin (5, 10, 20 µM) enhanced the expression and activation of ERα/β and Akt phosphorylation and inhibited H_2_O_2_-induced cell injury and apoptosis [91].

#### 3.1.7. Chalcones and Dihydrochalcones

Chalcones occur in tomatoes, licorice, shallots and bean sprouts, while dihydrochalcones are present in apples [17].

Several studies reported the important role of chalcones against I/R injury. Isolated rat hearts, pretreated with licochalcone D (1 μg/mL) or licochalcone B (0.5, 1 μg/mL) and then subjected to I/R, showed decreased apoptosis, decreased expression of pro-inflammatory cytokines, reduced cells necrosis and increased expression of anti-oxidant enzymes. The authors supposed that licochalcone D exerted these effects through the activation of Akt and the block of NF-κB/p65 and p38 MAPK pathways [92,93]. Isoliquiritigenin treatment (100 μM) of cardiomyocytes isolated from hearts with I/R, activated the AMP-activated protein kinase (AMPK) and MAPKs signaling pathways while reduced the mitochondrial potential and cardiac ROS levels [94]. It has been reported that safflor yellow A (SYA), a chalcone extracted from *Carthamus tinctorius*, and hydroxysafflor yellow A (HSYA) protected H9c2 and primary cultured neonatal rat cardiomyocytes exposed to A/R. The treatment with SYA (40, 60, 80 nM) or HSYA (20 µM) before A/R injury increased the levels of anti-oxidant enzymes and decreased cells necrosis and apoptosis through the up-regulation of HO-1 expression [95,96].

Another study investigated the in vitro effect of L6H9, a novel chalcone derivative, in the pathogenesis of diabetic cardiomyopathy (DCM). The treatment with L6H9 (10 μM) before incubation with glucose decreased the levels of pro-inflammatory cytokines, pro-apoptotic proteins and ROS in H9c2 cardiomyocytes. In addition, L6H9 increased the expression of Nrf2 and Nrf2-downstream anti-oxidant genes and inhibited fibrosis and hypertrophy [97].

Dludla et al. performed a study on the effects of a popular South African herbal tea, rooibos, produced from the leaves and stems of *Aspalathus linearis*, on diabetic cardiomyopathy (DCM). The authors evaluated the effects of an aqueous extract of fermented rooibos (FRE), containing the chalcone aspalathin and the flavonoids isoorientin, orientin, quercetin-3-*O*-robinobioside, in cardiomyocytes isolated from STZ-induced diabetic rats. Pretreatment of cardiomyocytes with FRE (1 and 10 µg/mL) protected cardiomyocytes against experimentally induced oxidative stress and ischemia [98]. Another study demonstrated that aspalathin, found in *Aspalathus linearis* (rooibos), protected H9c2 cardiomyocytes from chronic hyperglycemia-mediated apoptosis, by inhibiting the loss of mitochondrial membrane potential and ROS production and by increasing anti-oxidant enzymes [99].

### 3.2. Phenolic Acids

Phenolic acids are derivatives of benzoic acid and cinnamic acid and include the hydroxybenzoic acids (protocatechuic acid, and gallic acid) and the hydroxycinnamic acids (caffeic acid, ferulic acid, *p*-coumaric acid, and sinapic acid). The formers are found in few edible plants, while the laters are found in fruits, coffee, and cereal grains [17]. Phenolic acids were able to protect cardiomyocytes against H_2_O_2_-induced injury [100,101,102,103,104]. Gui-ling-gao (GLG, also known as turtle jelly) is a traditional food in Southern China and Hong Kong. Li et al. revealed nine compounds in GLG using mass spectrometry: caffeoylquinic acid, 5-*O*-caffeoylshikimic acid, astilbin, 3,4-dicaffeoylquinic acid, 4,5-dicaffeoylquinic acid, neoisoastilbin, isoastilbin, engeletin and 3,5-dicafeoyylquinic acid. GLG (1.372 mM) had the ability to scavenge free radicals and to abolish hemolysis, an index of oxidative damage. GLG increased cell survival and reduced apoptosis in H9c2 cardiomyocytes exposed to H_2_O_2_ [100]. *Castanea sativa* Mill. (CSM)-bark extract contains tannins and phenolic compounds, including ellagic acid, gallic acid, and 4 ellagitannins (vescalin, castalin, vescalagin and castalagin). Pretreatment with CSM-bark extract (50, 100 µg/mL) of neonatal rat cardiac myocytes for 24 h prior to H_2_O_2_ exposure protected cells from oxidative stress-induced injury [101]. *Phellinus linteus* is a medicinal mushroom rich of a phenolic compound, the hispidin. H9c2 cardiomyocytes treated with hispidin (30 µM) prior to H_2_O_2_ exposure, showed increased cell viability, reduced cells necrosis and ROS generation. In addition, hispidin decreased H_2_O_2_-induced apoptosis through the activation of Akt/GSK3β and MAPKs [102]. Neonatal rat cardiac myocytes (RCMs) pretreated with methyl gallate (50 µM) for 30 min prior to exposure to cobalt or H_2_O_2_, showed reduced apoptosis and ROS levels [103]. Ku et al. compared the in vitro effect of caffeic acid (CA) and pyrrolidinyl caffeamide (PLCA), a derivative of CA. HL-1, a mouse atrial cardiomyocyte cell line, was pretreated with CA and PLCA (3 μM) for 1 h before H_2_O_2_ administration. PLCA reduced the production of ROS and increased cells viability more than CA. In addition, PLCA treatment preserved the expression of anti-oxidant enzymes in cardiomyocytes [104].

Song et al. demonstrated the cytoprotective effect of ferulic acid (1, 5, or 10 µg/mL) in hepatocytes and cardiomyocytes against HG-induced injury and oxidative stress [105].

Atale et al. analyzed the effect of gallic acid-enriched methanolic *Syzygium cumini* pulp extract (MPE) on cardiotoxicity induced by malathion, an organophosphate pesticide. MPE treatment (20 µg/mL) restored the integrity of extra cellular matrix components and reduced the malathion-induced oxidative stress in H9c2 cardiomyocytes [106].

The beneficial effects of caffeic, chlorogenic and rosmarinic acids against DOX-induced toxicity were investigated in neonatal rat cardiomyocytes. Hydroxycinnamic acids (100, 200 µM) attenuated DOX-induced cell damage and the iron-dependent DOX-induced lipid peroxidation of microsomal and mitochondrial membranes [107].

Danshensu (3-(3,4-dihydroxyphenyl)-2-hydroxypropanoic acid) is the major water-soluble active component of Danshen, the dried root of the traditional Chinese herb *Salvia miltiorrhiza*, and it has been analyzed for its cardioprotective effects. Treatment with danshensu (10 µM) during reperfusion protected H9c2 cardiomyocytes from I/R-induced apoptosis [108]. Similarly, isolated rat hearts, perfused with danshensu (1, 10 µM) for 20 min and then subjected to I/R, showed reduced cells necrosis and ROS production and increased activity of anti-oxidant enzymes. These effects were achieved by the activation of Akt/ERK1/2/Nrf2 signaling pathways [109].

### 3.3. Lignans

Lignans (ecoisolariciresinol, matairesinol, medioresinol, pinoresinol, and lariciresinol) are found in high concentration in linseed and in minor concentration in algae, leguminous plants, cereals, vegetables, and fruits [17].

Two studies investigated the transduction pathways involved in the cytoprotective effect of (−) schisandrin B (Sch B), the most abundant dibenzocyclooctadiene lignan in *S. chinensis* (Turcz.) Baill, in H9c2 cardiomyocytes. The treatment of H9c2 cardiomyocytes with (−)Sch B (15 or 20 µM) activated the MAPKs, increased the nuclear expression of Nrf2 and reduced the H/R-induced apoptosis and inflammation [110,111]. Chang et al. showed that the treatment with deoxyschizandrin (DSD, 1 µM) and schisantherin A (STA, 1 µM) reduced apoptosis in cardiac cells subjected to I/R [112].

Cho et al. investigated the effect of syringaresinol in modulating HIF-1 (hypoxia inducible factor-1) during H/R injury. Syringaresinol (25 µM) protected H9c2 cardiomyocytes against H/R induced-cells damage, and reduced apoptosis. In addition, syringaresinol suppressed the expression of HIF-1 but stimulated the nuclear expression and activation of FoxO3, which decreased the expression of ROS and increased the expression of anti-oxidant enzymes [113].

Su et al. evaluated the in vitro effect of sesamin in DOX-induced citotoxicity in H9c2 cardiomyocytes. Treatment with sesamin (40 µM) increased cells viability, reduced the release of free radicals and the mitochondrial damage induced by DOX [114]. Zheng et al. demonstrated that sesamin treatment reduced the Ang II-induced apoptotic rate and ROS production in H9c2 cardiomyocytes [115].

### 3.4. Stilbenes (Resveratrol)

The main dietary source of stilbenes is resveratrol (3,5,4′-trihidroxystilbene) from red wine, grapes, berries, plums, peanuts, and pine nuts [17]. Many studies investigated the protective effects of resveratrol against H_2_O_2_-induced damage in cardiomyocytes. Resveratrol treatment (10–50 µM) protected H9c2 cardiomyocytes from H_2_O_2_-induced cell injury, apoptosis and autophagy and reduced ROS generation through activation of SIRT1 and mitochondrial biogenesis signaling pathways [116,117,118]. Movahed et al. also demonstrated that pretreatment with resveratrol (30 µM) prevented necrosis and the reduction of anti-oxidant enzymes activity in H_2_O_2_-exposed adult rat cardiomyocytes [119]. In addition, resveratrol treatment (50 µM) was effective in protecting rabbit ventricular myocytes against the oxidative stress-induced arrhythmogenic activity and Ca^2+^ overload, through the reduction of ROS production and inhibition of Ca^2+^/calmodulin-dependent protein kinases II (CaMKII) [120]. A study by Chen et al. demonstrated the protective effect of resveratrol against apoptosis induced by hypoxia in H9c2 cardiomyocytes. Resveratrol (20 µM) increased SIRT1 expression, leading to inhibition of forkhead box protein O1 (FoxO1) expression and activity [121].

Several studies showed the cardioprotective role of resveratrol in cardiomyocytes exposed to I/R injury. Pretreatment with resveratrol (20 µM) protected neonatal rat ventricular cardiomyocytes from I/R-induced oxidative injury, apoptosis, necrosis and mitochondrial dysfunction through the induction of SIRT1 which modulated MAPKs signaling [122]. Resveratrol was effective in attenuating the cardiotoxicity induced by chemotherapeutic drugs, such as DOX and arsenic trioxide, and by anti-retroviral agents, such as azidothymidine (AZT). Danz et al. observed that resveratrol (10 µM) inhibited DOX-induced ROS generation and necrosis through the activation of SIRT1 pathway in neonatal rat ventricular myocytes [123]. Similarly, Gao and colleagues reported that pretreatment with resveratrol (3µM) attenuated the mitochondrial ROS generation, apoptosis and necrosis induced by AZT in human primary cardiomyocytes [124]. Zhao et al. investigated the protective effect of resveratrol against arsenic trioxide (As_2_O_3_)-induced cardiotoxicity in H9c2 cardiomyocytes. They demonstrated that pretreatment with resveratrol (10 µM) reduced cells apoptosis and necrosis, increased cell viability and inhibited the generation of ROS and the intracellular Ca^2+^ mobilization induced by As_2_O_3_ [125].

Resveratrol was also able to protect primary cultures of neonatal rat cardiomyocytes against HG-induced apoptosis through the AMPK pathway. Resveratrol (50 µM) inhibited apoptosis and ROS production and increased the activity of anti-oxidant enzymes [126]. Das et al. demonstrated that the treatment of human and murine cardiomyocytes with resveratrol (100 μM) reduced the iron-induced oxidative stress by decreasing the levels of free radicals. The authors observed that the inhibition of oxidative stress was dependent on the increase of SIRT1 and on the reduction of FoxO1 expression [127].

Several studies investigated the in vitro effects of resveratrol derivatives. The effect of polydatin, a resveratrol glucoside, against phenylephrine induced-cardiac hypertrophy was investigated in neonatal rat cardiomyocytes. Polydatin (50 µM) induced an anti-hypertrophic effect by decreasing ROS production and by inhibiting the RhoA/ROCK signaling pathway, that play a role in mediating the development of cardiac hypertrophy and heart failure [128]. Recently, Feng et al. investigated the effect of an analog of resveratrol, bakuchiol (BAK), a monoterpene phenol isolated from the seeds of *Psoralea corylifolia* (Leguminosae), against the I/R-induced oxidative damage. Pretreatment with BAK (1 µM), administered 5 min before I/R, protected isolated rat hearts from I/R through the activation of the SIRT1 pathway. BAK improved cardiac function, reduced apoptosis and increased anti-oxidant enzymes [129].

### 3.5. Other Polyphenols

#### 3.5.1. Curcumin

Curcumin (1,7-bis-(4-hydroxy-3-methoxyphenyl)-1,6-heptadiene-3,5-dione) is another polyphenol compound and a member of the curcuminoid family. It is found in turmeric, a spice produced from the rhizome of *Curcuma longa* [17].

Several studies investigated the protective effect of curcumin against the oxidative stress in cardiomyocytes. Pretreatment of HL-1 cells with curcumin (5 μM) reduced Ang II-induced cardiomyocytes hypertrophy, oxidative stress and apoptosis [130]. Kim et al. showed that the curcumin (10 µM) pretreatment reduced the cardiotoxicity caused by TNF-α, peptidoglycan or H/R in rat cardiomyocytes [131]. Curcumin also attenuated the I/R-induced toxicity through SIRT1 pathway [132]. Xu et al. analyzed the protective role of curcumin against A/R-induced mitochondrial injuries in rat hearts. Curcumin (1 µM) restored the mitochondrial respiratory activity, inhibited the lipoperoxidation, protein carbonylation and cells apoptosis [133]. Treatment of primary cultures of neonatal rat cardiomyocytes with curcumin (10 µM) reduced the glucose-induced toxicity by decreasing the apoptotic rate and ROS production. Curcumin reduced the toxicity through the NADPH-mediated oxidative stress and PI3K/Akt pathway [134].

Several studies investigated the cardioprotective effects of curcumin derivatives. Nehra et al. evaluated the effect of nanocurcumin after hypoxia. H9c2 cardiomyocytes treated with nanocurcumin (50 µM), compared to cells treated with curcumin, showed reduced hypertrophy and apoptosis [135]. In addition, nanocurcumin (50 ng/mL) inhibited the hypertrophy, reduced necrosis and ROS generation, restored the mitochondrial membrane potential and glucose transporters and increased ATP levels in primary human ventricular cardiomyocytes (HVCM) [136]. The treatment of hearts with AID (alpha-interacting domain of the l-type Ca^2+^ channel) peptide tethered nanoparticles containing 25 mg of curcumin (NP-C-AID) or resveratrol (NP-R-AID) reduced cells necrosis, ROS generation and mitochondrial membrane potential in response to I/R injury. However, only NP-C-AID reduced the oxidative stress by balancing the GSH/GSSG ratio, and the muscle damage [137]. The effects of monocarbonyl analogues of curcumin (MACs) with high stability was evaluated in I/R-induced toxicity and injuries. Among these MACs, the curcumin analogue 14p (10 µM) reduced the toxicity by blocking the oxidative stress, apoptosis and by activating the Nrf2 pathway in H9c2 cardiomyocytes [138].

#### 3.5.2. Olive Oil Polyphenols

Olive oil is a source of at least 30 phenolic compounds. The three phenolic compounds found in highest concentration in olive oil are oleuropein, hydroxytyrosol and tyrosol [139].

Bali et al. compared the effects of the ethanolic and methanolic extracts of olive leaves with the effects of oleuropein, hydroxytyrosol, and quercetin, as a positive control, in H9c2 cardiomyocytes exposed to 4-hydroxy-2-nonenal (HNE), which induced oxidative damage. H9c2 cardiomyocytes were pretreated with each compound (0.1, 10 μg/mL) and then exposed to HNE. Both extracts of olive leaves and the other compounds inhibited cells apoptosis, blocked ROS production, counteracted mitochondrial dysfunction. The ethanolic extract, that contained large amounts of oleuropein, hydroxytyrosol, verbascoside, luteolin, and quercetin, exerted a better protection against HNE-induced cardiotoxicity than the methanolic extract or each phenolic compound [140].

#### 3.5.3. Salvianolic Acid

Salvianolic acid B (SalB), derived from the Chinese herb *Salvia miltiorrhiza*, was investigated against TNF-α-induced MMP-2 up-regulation and oxidative stress in human aortic smooth muscle cells (HASMCs). Pretreatment with SalB (0.1, 1, 10 µM) inhibited the TNF-α-induced increase of ROS production. In addition, the polyphenol suppressed the TNF-α-induced MMP-2 enzymatic activity [141]. SalB (5, 10 µM) was also able to protect human aortic endothelial cells (HAECs) from oxidized-low density lipoprotein (ox-LDL) induced oxidative injury via inhibition of ROS production [142].

#### 3.5.4. Silymarin and Silibinin

Silymarin (SM) is a standardized extract from the seeds of *Silybum marianum L. Gaertneri*, and the main constituents are silibinin, and other flavonolignans (dehydrosilibinin, silychristin and silydianin). Silymarin (0.50 mg/mL) showed ROS radical scavenging activities. Silymarin (0.50 mg/mL) improved mitochondrial functions, reduced NO levels, protein carbonyl content and lipid peroxidation and increased anti-oxidant and Kreb’s cycle enzymes in cardiac mitochondria exposed to copper-ascorbate [143].

Silibinin (50–200 μM) reduced cellular damage after H_2_O_2_-exposure in H9c2 cardiomyocytes. In addition, the treatment with silibinin (100–200 μM) inhibited MAPKs and Akt activation in phenylephrine-induced hypertrophic H9c2 cardiomyocytes [144]. Gabrielova et al. demonstrated that the treatment of rat neonatal cardiomyocytes with 2,3-dehydrosilybin (DHS) (10 µM) attenuated the H/R-induced damage by decreasing the generation of ROS and protein carbonyls [145].

### 3.6. Combination of Polyphenols and Comparative Studies

Esmaeili and Sonboli demonstrated the anti-oxidant and free radical scavenging activities of *Salvia brachyantha* extract, which contains phenolic and flavonoid compounds such as rosmarinic acid, caffeic acid, luteolin, gallic acid, rutin and catechin. The pretreatment of H9c2 cardiomyocytes with the *Salvia brachyantha* extract (50–100 μg/mL) reduced the production of ROS, increased anti-oxidant enzymes and prevented cells apoptosis induced by xanthine/XO [146].

Chen et al. analyzed the cardioprotective role of the total flavonoids from *Clinopodium chinense* (Benth.) O. Ktze (TFCC), against the adverse effects of DOX treatment in H9c2 cardiomyocytes. H9c2 cardiomyocytes treated with TFCC (25 μM) showed increased levels of anti-oxidant enzymes and decreased cells necrosis and apoptosis and ROS production. Furthermore, TFCC blocked DOX-induced over-expression of p53 and regulated the phosphorylation of MAPKs, Akt and PI3K [147].

Chang et al. evaluated and compared the oxidant scavenging capacity of five flavonoids (wogonin, baicalin, baicalein, catechin and procyanidin B2) and their protective efficacy in a chick cardiomyocytes model. Catechin and procyanidin B_2_ showed the best scavenging capacity for DPPH (1,1-diphenyl-2-picryhydrazyl) and superoxide radicals. Only baicalein exhibited a significant hydroxyl radical scavenging potency. The cardiomyocytes were treated with three different treatment protocols during I/R. The cardiomiocytes were exposed to flavonoids (25 μM) before and during I/R (chronic treatment) or during I/R or during reperfusion phase. The administration of all flavonoids, except for wogonin, reduced apoptosis in chronic treatment. Baicalein, procyanidin B_2_, and catechin reduced apoptosis when flavonoids were administered only during I/R. In addition, only baicalein and procyanidin B_2_ treatment during reperfusion phase reduced cells apoptosis. Flavonoids had different radical scavenging capacities and protective effects against I/R injury depending on the timing of treatment [148].

Akhlaghi et al. analyzed the effects of several flavonoids (all at 25 µM) in H9c2 cardiomyocytes exposed to I/R injury. The pretreatment with catechin improved cells viability, while cyanidin showed a mild protection. However, only the pretreatment of H9c2 cardiomyocytes with quercetin and EGCG reduced the oxidative stress [149].

Mechanisms of action of in vitro effects of polyphenols to counteract the oxidative stress-induced cardiotoxicity are summarized in Table 1.

## 4. Bioavailability of Polyphenols: Is the Effective Polyphenols Dose Feasible In Vivo?

Although in vitro studies reported that polyphenols exert strong anti-oxidant activity, their beneficial effects in humans remains poor, because of their low bioavailability. Bioavailability is the quantity of a compound that is absorbed and metabolized within the human body after dietary intake and it is measured commonly with the maximum plasma concentration (Cmax) reached after the intake. In fact, in order to produce in vivo effects, a compound must enter the circulation and reach the tissues, in the native or metabolized form, in a sufficient dose to exert biological activity [17]. After dietary intake, polyphenols are metabolized and then the metabolites induce the physiological effects observed in in vivo models [150,151]. The doses of polyphenols employed in vitro usually range between μmol/L and mmol/L, but the concentrations of metabolites in the plasma are of only nmol/L [150]. Endogenous factors affect the bioavailability of polyphenols, such as their metabolism in the gastrointestinal tract and liver, their binding on the surfaces of blood cells and microbial flora in the oral cavity and gut, and regulatory mechanisms that prevent the toxic effects of high polyphenols levels on mitochondria or other organelles [152]. Furthermore, polyphenols are often used as aglycones or conjugates to sugar and not as their active metabolites in in vitro studies [150].

Polyphenols can interact with several substances that influence their bioavailability. For example, polyphenols can bind proteins and metal cations that modify their absorption [153]. Dietary factors can affect the bioavailability of polyphenols, such as food matrix and food preparation techniques [154]. The rate and extent of intestinal absorption and the metabolites in the plasma mainly depend on the chemical structure of polyphenols. Polyphenols show different bioavailability and it has been estimated that the plasma concentration of polyphenols and total metabolites reached after an intake of 50 mg of aglycones ranged from 0 to 4 µM. In addition, the half-lives of polyphenols in human plasma are in the range of few hours and depend on the food source [155]. However, in order to assess the real in vivo potential of polyphenols it is essential to know not only their plasma concentration, but the concentration of the active metabolites in the target tissues [155]. Thus, it would be necessary to employ the active metabolites of polyphenols in in vitro studies [151]. Accordingly, although it is very difficult, it would be important to analyze the concentration of polyphenols in a specific tissue [150]. A limited number of studies detected the concentration of polyphenols in different tissues of mice and rats, including endothelial cells and heart, after administration of the compounds. The tissue concentrations are different depending on the type of tissue and the dose administered, but tipically range from 30 to 3000 ng aglycone equivalents/g tissue [13]. Unfortunately, it is much more difficult to evaluate the tissue concentrations of polyphenols in humans and in fact the studies are very scarce [150]. Overall, a better knowledge of this issue is needed.

Despite the limit of the in vitro studies, anti-oxidative effects of polyphenols have been reported in in vivo experimental models.

## 5. In Vivo Effects of Polyphenols Against Oxidative Stress-Induced Cardiotoxicity

### 5.1. Flavonoids

#### 5.1.1. Flavonols

Several studies showed the in vivo cardioprotective effects of flavonols against DOX-induced cardiotoxicity. The quercetin (100 or 15 mg/kg) pretreatment of mice and rats treated with DOX improved cardiac function, blocked ROS generation and lipid peroxidation and increased anti-oxidant enzymes [48,156]. Isorhamnetin (5 mg/kg, i.p.), kaempferol (10 mg/kg, i.p.) and dihydromyricetin (125–500 mg/kg) administration reduced cardiomiocytes necrosis and apoptosis, ROS production and lipid peroxidation and increased the production of anti-oxidant enzymes in in vivo models exposed to DOX [49,50,51].

Flavonols effects were analyzed also in rats underewent I/R injury. Rats treated with kaempferol (15 mM) recovered cardiac function, reduced infarct size and cardiomyocytes apoptosis [157].

Astragalin (kaempferol-3-*O*-glucoside) pretreatment (10 µM) showed cardioprotective effects in rats via its anti-oxidative, anti-apoptotic, and anti-inflammatory activities [158]. The in vivo effects of 3′,4′-dihydroxyflavonol (DiOHF) was investigated in sheep. The in vivo administration of DiOHF (5 mg/kg) after I/R injury reduced ROS generation, neutrophil accumulation in coronary microvessels, left ventricular diastolic pressure (LVDP) and infarct size, increased the left anterior descending (LAD) coronary artery blood flow, the total plasma nitrate and nitrite and restored the myocardial function [55]. The myocardial I/R injury was blocked by NP202, a novel pro-drug of DiOHF, through the activation of MAPKs signaling in sheep [47].

A study by Bhandary et al. investigated the preventive effects of rutin, quercetin and various isoflavones (biochanin A, daidzein, genistein) in an ex vivo model of I/R injury. They perfused rat hearts with isoflavones (10 µM), quercetin (10 µM), and rutin (50 µM) before ischemia and during the reperfusion time. Only rutin possessed the ability to attenuate cardiac I/R-associated hemodynamic alterations [159]. In addition, rutin increased anti-oxidant molecules and reduced cells necrosis and apoptosis and overall cardiac dysfunction in rats exposed to sodium fluoride-induced oxidative stress [160].

The isoproterenol injection induced cardiotoxicity in rats. The co-treatment with vincristine (25 mg/kg) and quercetin (10 mg/kg) attenuated the cardiotoxicity induced by isoproterenol [161].

Guo et al., employing a mouse model with transverse aortic constriction (TAC), demonstrated that taxifolin, a dihydroquercetin derivative decreased ROS levels, attenuated the pressure overload, blocked the cardiac remodeling, ventricular dysfunction and fibrosis [56].

#### 5.1.2. Flavones

Several studies demonstrated the in vivo cardioprotective role of flavones against I/R. In murine and rat models baicalein (30 mg/kg), apigenin (5 mg/kg) and vitexin (6, 3, 1.5 mg/kg) reduced I/R injury-induced infarct size, apoptosis, pro-inflammatory cytokines and oxidative stress [162,163,164]. Employing diabetic rats subjected to myocardial I/R, it has been assessed the protective effects of luteolin or breviscapine, a flavonoid extracted by *Erigeron breviscapus*. Luteolin (100 mg/kg/day intragastrically) or breviscapine (60 mg/kg orally) reduced the ROS production [165,166]. Luteolin also improved left ventricular function and cardiac tissue viability [165]. In addition, breviscapine reduced the expression of ICAM-1 in rat myocardium, the inactivation of ATPase and associated ionic disturbances [166].

The protective effect of apigenin in isolated rat heart suffered from A/R was also demonstrated. Rats intraperitoneally injected with apigenin (4 mg/kg) showed reduced cells death and infarct size [58].

#### 5.1.3. Flavan-3-Ols

Several studies reported that the administration of green tea extract (GTE) (200 mg/kg or 400 mg/kg, prior to I/R injury) to rats reduced the myocardium damage, infarct size and apoptosis and increased the anti-oxidant enzymes [167,168]. The anti-oxidant properties of flavan-3-ols were also evaluated in DOX-exposed in vivo models. Oral pretreatment for 30 days with GTE (100, 200 and 400 mg/kg) or procyanidins (150 mg/kg daily) decreased the cardyomyocytes death and increased anti-oxidant enzymes in rat models exposed to DOX [169,170].

Sheng et al. reported the protective effects of EGCG in cardiac hypertrophy induced by abdominal aortic constriction in rats. They showed that EGCG (50 and 100 mg/kg) administered intragastrically to rats for 6 weeks, decreased myocardium damage and ROS generation. The highest doses of EGCG improved histological changes in the heart tissue, inhibited fibrosis and myocytes apoptosis [67]. Using the same rat model, the authors evaluated the effect of EGCG and other traditional anti-hypertrophic therapeutic agents on telomere dysfunction mediated apoptotic signal. They demonstrated that treatment for 6 weeks with EGCG (50 and 100 mg/kg) or quercetin (100 mg/kg) reduced heart weight indices and cardiac myocytes apoptosis in the hypertrophic myocardium [171].

Another investigation reported the effect of grape seed proanthocyanidins (GSP) against cadmium (Cd), a toxic heavy metal that induced oxidative stress and cardiotoxicity. In this study, GSP pretreatment (100 mg/kg) reduced Cd-induced disruption of cardiac myofibrils, cardiomyocytes necrosis and apoptosis, production of pro-inflammatory cytokines and increased the levels of anti-oxidant enzymes in rats [172]. Wang et al. demonstrated that GSP (195 mg/kg, intragastric administration) treatment prevented deoxycorticosterone (DOCA)-salt-induced cardiovascular remodeling and endothelial dysfunction in mice, in part through the involvement of oxidative stress [173].

#### 5.1.4. Anthocyanins

Liu et al. reported the effects of blueberry anthocyanins-enriched extracts (BAE) on cyclophosphamide (CTX)-induced cardiac toxicity in rats. BAE (20 and 80 mg/kg daily by gavage) treatment attenuated the CTX-induced cardiac injury through its anti-inflammatory and anti-oxidant proprieties. BAE decreased mean arterial blood pressure and myocardial leukocyte infiltration, increased heart rate and improved cardiac dysfunction, and reduced LV hypertrophy and fibrosis. Rats treated with BAE showed a decrease expression of pro-inflammatory cytokines, an increase of anti-inflammatory cytokines and anti-oxidant enzymes [174].

#### 5.1.5. Flavanones

Two studies reported the cardioprotective role of the flavanone glycoside hesperidin on I/R-induced arrhythmias in an in vivo rat model. Rats were administered with hesperidin (100 mg/kg, p.o. for 15 days) and then subjected to I/R. The administration of hesperidin induced antiarrhythmic effects, a reduction of inflammation, oxidative stress and cardiomyocytes apoptosis [175,176]. A similar study was conducted with naringin. Naringin was administered *per os* (40, 80 mg/kg, daily) to rats for 14 days and then I/R injury was induced by coronary artery occlusion. Naringin restored I/R injury, as demonstrated by the normalization of cardiac injury markers, by the increased activity of anti-oxidant enzymes, by the reduction of cells apoptosis, infarct size and inflammation [177].

The protective effect of hesperetin against DOX-induced oxidative stress and DNA damage was analyzed in rat heart. Rat, treated with DOX and hesperetin (50 and 100 mg/kg b.w., p.o. by gavage for 5 consecutive days in a week), showed decreased ROS production and cells apoptosis [178]. Hesperetin (30 mg/kg, p.o.) and hesperidin (200 mg/kg, p.o.) administration to murine and rat models inhibited the cardiac remodeling and fibrosis, oxidative stress and apoptosis induced by pressure overload and isoproterenol [179,180].

In addition, Elavarasan et al. also demonstrated that the anti-oxidant activity of hesperidin protected cardiac tissue of aged rats against age-related increase in oxidative stress. Hesperidin (100 mg/kg/day, p.o. for 90 days) increased the activity of anti-oxidant enzymes [181].

The cardioprotective role of hesperidin and naringin on hyperglycemia-induced oxidative damage in HFD/streptozotocin (STZ)-induced diabetic rats has been reported by Mahmoud et al. Diabetes was induced and then rats were treated with hesperidin or naringin (50 mg/kg, p.o.) daily for 4 weeks. Oral administration of hesperidin and naringin prevented diabetic complications and increased the activities of anti-oxidant enzymes in experimental diabetic rats [182].

#### 5.1.6. Isoflavones

In vivo effects of calycosin-7-*O*-β-D-glucoside (CG) on I/R injury were investigated by Ren et al. Pretreatment of rats with CG (30 mg/kg i.v.) 30 min before the ligation of the left anterior descending (LAD) coronary artery was able to improve cardiac function, to decrease infarct size and to enhance the activity of SOD. CG alleviated I/R injury through the activation of the PI3K/Akt pathway and the inhibition of apoptosis [183].

Gang et al. investigated the in vivo effect of puerarin in Ang II-induced cardiac hypertrophy. Puerarin (100 and 200 mg/kg, gavage) reduced cardiac hypertrophy induced by Ang II in mice [90]. The effect of puerarin on severe burn-induced acute myocardial injury in rats was assessed by Liu et al. Groups of adult Wistar rats were subjected to a 30% TBSA (total body surface area) full-thickness dermal burn and resuscitated with an intraperitoneal injection of 4 mL/kg/TBSA of lactated Ringer’s solution with or without puerarin (10 mg/kg). Results showed that puerarin protected cardiomyocytes from severe burn-induced ultrastructural modifications and death [184].

#### 5.1.7. Chalcones and Dihydrochalcones

Zhong et al. evaluated the in vivo effects of the chalcone derivative L6H9 against STZ-induced diabetes in C57BL/6 mice. L6H9 (20 mg/kg by gavage) protected multiple organs in diabetic mice. In particular, the heart of mice treated with L6H9 did not show structural abnormalities and fibrosis. These mice showed lower levels of ROS, cytokines, and apoptosis [97].

Several studies investigated the in vivo effects of chalcones against I/R. Treatment with licochalcone C (2.0 mM) increased LVDP, levels of anti-oxidant enzymes and decreased cardiomyocytes mitochondrial injury, necrosis and apoptosis in rats [185]. Another study showed the effects of intraperitoneal injection of Cl-chalcone (50 μg/kg) and F-chalcone (100 μg/kg) in albino rats with I/R. Rat hearts treated with Cl-chalcone and F-chalcone showed limited infarct size and high levels of anti-oxidant enzymes [186]. A study reported the protective effect of rooibos extract against myocardial I/R injury. They showed that the aortic output recovery after reperfusion was improved in hearts isolated from male Wistar rats supplemented with aqueous rooibos extract for 7 weeks when compared to that supplemented with green tea (*Camellia sinensis*) extract [187].

### 5.2. Phenolic Acids

Ku et al. analyzed the response of pyrrolidinyl caffeamide (PLCA) against I/R-induced oxidative stress in rats. PCLA administration (1 mg/kg i.p.) reduced the levels of troponin and lipid peroxidation, improved cardiac functions and attenuated the myeloperoxidase (MPO) activity, a marker of neutrophil accumulation [104].

Tang et al. investigated the effect of danshensu in spontaneously hypertensive rats (SHR). Danshensu (10 mg/kg/day i.p.) decreased the HW/BW index, prevented the increase of blood pressure, and reduced arrhythmias in rats exposed to I/R [188]. In addition, danshensu possessed a cardioprotective effect against I/R injury when given to rats during reperfusion (30 and 60 mg/kg, for 3 h) [108].

The protective effects of the preparation Shenge, composed by 1:1 ratio of puerarin and danshensu, has been evaluated on acute ischemic myocardial injury in rats. Rats were subjected to LAD coronary artery occlusion and were injected intravenously with Shenge (30, 60, or 120 mg/kg b.w.) 15 min later. Shenge was able to improve electrocardiographic changes, the size of ischemic area and anti-oxidant activities induced by the acute myocardial ischemia [189].

### 5.3. Lignans

Several studies analyzed the cardioprotective effects of Schisandrin (Sch) B against DOX-induced oxidative stress. The administration of Sch B (25, 50 and 100 mg/kg) to mice and rats reduced cardiomyocytes apoptosis and necrosis in left ventricle and the activity of MMPs induced by DOX [190,191]. The pretreatment with Sch B (100 mg/kg) significantly increased the levels of GSH redox cycling enzymes [190]. Su et al. also evaluated the in vivo effect of sesamin in DOX-induced cardiotoxicity in rats. The treatment of rats with sesamin (20 mg/kg, orally, for 10 consecutive days) reduced the DOX-induced toxicity through the normalization of the electrocardiography and the decrease of histopathological changes [114]. Daily sesamin treatment (80 or 160 mg/kg, by gavage) in SHR for 16 weeks improved LV hypertrophy and fibrosis. The treatment reduced the systolic blood pressure, enhanced cardiac total anti-oxidant capabilities and TGF-β1 expression [192].

Chen et al., employing a mice model of myocardial infarction (MI) established by a permanent ligation of the left anterior descending (LAD) coronary artery, reported that the MI mice treated with Sch B (80 mg/kg) showed increased survival rate, improved heart function and decreased infarct size [111].

Chiu et al. demonstrated that Sch B also exerted a protective role against myocardial I/R injury through the activation of ERK pathway in rats. Sch B (1.2 mmol/kg, intragastric) decreased LDH leakage and increased GSH levels [110].

The effects of magnolol, extracted from *Magnolia officinalis*, deoxyschizandrin (DSD) and schisantherin A (STA) was analyzed in rats subjected to I/R. Magnolol (10 mg/kg, i.p.), DSD (40 µmol/kg, i.v.) and STA (40 µmol/kg, i.v.) reduced infarct size, arrhythmias and improved I/R-induced myocardial dysfunctions [112,193].

### 5.4. Stilbenes (Resveratrol)

Resveratrol was able to prevent the development of oxidative stress in SHR. Treatment with resveratrol (2.5 mg/kg b.w., daily, by oral gavage for 10 weeks) reduced ROS production [119]. Dolinsky et al. reported that resveratrol (2.5 mg/kg b.w., daily, by oral gavage for 2 weeks) reduced the levels of cardiac HNE-protein adducts and the HNE adducts formation on liver kinase B1 (LKB1), leading to a reduction of left ventricular hypertrophy [194].

Zhao et al. investigated the effect of resveratrol in a mouse model of arsenic trioxide (As_2_O_3_)-induced cardiomyopathy in vivo. Pretreatment with resveratrol (3 mg/kg, i.v., on alternate days for three days 1 h before As_2_O_3_ administration) attenuated As_2_O_3_-induced structural and electrocardiographic abnormalities, increased increased anti-oxidant activity of mice treated with As_2_O_3_ [125].

Resveratrol exerted a beneficial effect in mice with diabetic cardiomyopathy induced by strepozotocin (STZ). Mice fed with a diet enriched with resveratrol (60 and 300 mg/kg/day, for 16 weeks) showed an attenuation of oxidative injury, a decreased cardiomyocytes apoptosis and an improved cardiac function. In addition, resveratrol increased the autophagic flux in diabetic mouse hearts [195].

Das et al. reported that the oral administration of resveratrol reduced the myocardial injury induced by iron. Mice treated with resveratrol decreased oxidative stress and myocardial fibrosis. These responses were achieved by the increase in SIRT1 expression and the reduction in FoxO1 expression [127].

Resveratrol and polydatin, a resveratrol glucoside, attenuated DOX-induced cardiotoxicity in rats. Resveratrol (10 or 15 mg/kg) reduced cardiac dysfunction, oxidative damage and apoptosis and increased anti-oxidant enzymes [196,197]. In addition, a synergistic effect of polydatin and vitamin C to reduce the cardiotoxicity of DOX was observed. The combined treatment (polydatin and vitamin C both 200 μmol/kg) improved electrocardiography, reduced ROS production, increased anti-oxidant activities, and improved the myocardial metabolism in rats [198].

The treatment with resveratrol (20 mg/kg, s.c. or 5, 15, 45 mg/kg/day for 10 days, by gavage) protected mice and rats against LPS and cisplatin-induced cardiotoxicity by restoring the intracellular redox status [199,200]. The anti-hypertrophic effect of polydatin or isorhapontigenin, a resveratrol analogue, was demonstrated in C57BL/6 mice subjected to transverse aortic constriction (TAC), a model of pressure-overload-induced cardiac hypertrophy in vivo. Polydatin (50 mg/kg, daily by gavage) reduced the TAC-induced cardiac hypertrophy [128].

### 5.5. Other Polyphenols

#### 5.5.1. Curcumin

Several studies investigated the effect of curcumin in I/R. Curcumin exerts a cardioprotective effect against I/R injury through the reduction of oxidative stress and mitochondrial dysfunction. Curcumin pretreatment (200 mg/kg) reduced the loss of cardiac mechanical work, the lipid peroxidation and the levels of inflammatory markers in rats [131,201]. In addition, curcumin reduced the cardiotoxicity induced by I/R injury through the SIRT1 pathway. In fact, the in vivo pretreatment of rats with curcumin increased the levels of SIRT1, anti-oxidant enzymes and reduced the cellular damage and necrotic and apoptotic cells [132]. Li et al. showed that the administration of curcumin analogue 14p (10 mg/kg) decreased the infarct size and myocardial apoptosis to the same extent as the high dose curcumin (100 mg/kg) in a mouse model of myocardial I/R [138]. In another study, the co-treatment of rats with curcumin (60 mg/kg) and isoprenaline reduced the percentage of isoprenaline-induced apoptotic and necrotic cells and restored the levels of anti-oxidants. Curcumin decreased the opening of mPTP induced by isoprenaline without modify mitochondria [202].

Diabetic cardiomyopathies are associated with high levels of membrane-bound protein kinase C (PKC). Treatment of STZ-induced diabetic rats with curcumin (100 mg/kg/day for 8 weeks) reduced cardiomyocyte hypertrophy, oxidative stress, myocardial fibrosis and left ventricular dysfunctions [203].

Imbaby et al. analyzed the cardioprotective effects of curcumin and nebivolol against cardiotoxicity induced by DOX therapy in rats. Oral administration of curcumin (200 mg/kg) attenuated DOX-induced cardiotoxicity. The treatment increased survival rate and anti-oxidant enzymes, decreased lipid peroxidation and histological alterations [204].

#### 5.5.2. Olive Oil Polyphenols

In several studies, Andreadou et al. demonstrated the cardioprotective effect of oleuropein against DOX. Oleuropein administration (100 or 200 mg/kg for 5 or 3 consecutive days) reduced cells damage and necrosis in rats induced by DOX [205]. In addition, the same authors investigated the effect of oleuropein in chronic DOX-induced cardiomyopathy in rats. The intraperitoneal administration of oleuropein (1000 or 2000 mg/kg for 14 days) improved heart contractility, reduced apoptosis and production of inflammatory cytokines, degenerative myocardial lesions and levels of nitro-oxidative compounds [206].

#### 5.5.3. Silymarin and Silibinin

Several studies analyzed the effect of silymarin against the cardiotoxicity induced by anti-cancer drugs. Silymarin protected rats against DOX-induced injury. It has been observed that silymarin (50 or 100 mg/kg) reduced myocardial and renal damage and NO levels [207,208]. Silymarin treatment (25, 50 or 100 mg/kg) reduced the serum levels of necrosis markers, enhanced anti-oxidant activities and attenuated the damage of mitochondria in mice and rats exposed to cisplatin or acrolein [209,210].

The protective effect of silibinin against arsenic-induced oxidative stress has been analyzed in rats. Silibinin administration (75 mg/kg) reduced the myocardial damage, the activity of heart mitochondrial enzymes, levels of plasma and cardiac lipids, while it up-regulated the levels of anti-oxidant enzymes in hepatic tissues. Thus, silibinin attenuated the mitochondrial damage and restored the heart function [211].

### 5.6. Combination of Polyphenols

Chen et al. evaluated the cardioprotection of flavonoids extracted from *Clinopodium chinense* (Benth.) O. Ktze (TFCC) against DOX treatment in rats. TFCC (20, 40 and 80 mg/kg) treatment induced the recovery of body and heart weights, balanced the levels of cardiac enzymes, inhibited ROS production and apoptosis and up-regulated the level of anti-oxidant enzymes [147].

Table 2 summarizes molecular mechisms of in vivo effects of polyphenols against the oxidative stress-induced cardiotoxicity.

**Figure 3:**
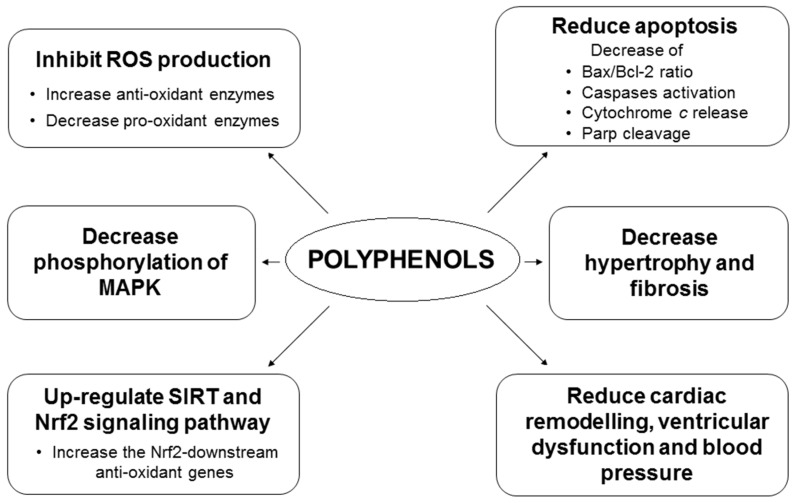
In vitro and in vivo effects of polyphenols in cardiovascular disease.

Overall, Figure 3 shows the in vitro and in vivo effects of polyphenols in cardiovascular disease.

## 6. Evidence from Human Studies

Although human studies are not the main focus of this review and have been reviewed elsewhere [153,212,213], a short section of results from clinical trials is reported. This section might help to understand whether in vitro and in vivo results obtained in experimental models can be mimicked in humans. Many human studies have investigated the potential effects of polyphenols, such as cocoa-, olive-, tea-, grape-contained polyphenols, in cardiovascular diseases [212]. Recently, it has been reported that resveratrol intake (10 mg resveratrol capsule, per day for 3 months) reduced LDL, platelet aggregation and improved endothelial function and vasodilation in 40 patients with coronary artery disease [214]. In another study, resveratrol (400 mg, daily, for one month) reduced the expression of ICAM-1, VCAM-1, IL-8 and mRNA of inflammatory and adhesion molecules in 44 healthy subjects. The inflammatory markers are related with atherosclerosis and oxidative stress. Thus, resveratrol intake could represents a preventive measure for the onset of atherosclerosis [215].

Zunino et al. investigated the effects of dietary grapes in a randomized, double-blind crossover study in 24 obese human subjects. The obesity is associated with a major risk of cardiovascular diseases. The authors reported that dietary grape powder supplementation (46 g, two times per day for 3 weeks) reduced the plasma concentration of LDL and increased the production of IL-1β and IL-6 in supernatants from lipopolysaccharide-activated peripheral blood mononuclear cells (PBMCs) [216]. Zern et al. observed that the grape powder supplementation (36 g, per day for 4 weeks) showed an improvement in plasma lipids, inflammatory cytokines, and oxidative stress in 24 pre- and 20 post-menopausal women. This trial suggested that grape powder can influence the expression of key risk factors for coronary heart disease in pre- and post-menopausal women [217]. Vaisman and Niv showed that red grape cell powder consumption (200 or 400 mg, per day for 12 weeks) improved the endothelial function, diastolic blood pressure and reduced oxidative stress without adverse effects in 50 subjects with pre-hypertension and mild hypertension [218]. However, several randomized placebo-controlled trials did not show differences between placebo and treated groups [212]. For example, in a double-blind, randomized crossover trial, Mellen and colleagues reported that the muscadine grape seed supplementation (1300 mg, per day for 4 weeks) did not improve endothelial function, blood pressure or did not change the plasma markers of cardiovascular risk in 50 subjects with increased cardiovascular risk [219].

Several studies reported the improvement of blood pressure after cocoa intake, in particular dark chocolate, in hypertensive subjects and with endothelial dysfunction. It has been reported that flavanol-enriched cocoa drink increased the levels of NO and NOS activity by improving the endothelial dysfunction. But some trials did not show this effects both in short treatments and in supplementation of one year with 27 g flavonoid-enriched chocolate [212].

Overall, the conclusions that may be drawn from clinical trials are much different from in vitro and in vivo studies. Indeed, results from human trials employing polyphenols showed a controversial response [212].

## 7. Limitations of the Polyphenols Studies

Overall, although in vitro studies were necessary to determine the polyphenols effective dose and their mechanism of action, they have some limitations. One of the major drawback of in vitro studies is the use of polyphenols in the aglycones form or in coniugation with sugar moieties and not the use of the active metabolites. In addition, the effective doses were much higher than the concentrations that can be reached in humans. Accordingly, the in vitro results must be carefully decoded.

The results obtained from in vivo studies also have some limitations. Indeed, the majority of the information regarding the metabolism and distribution of polyphenols in target tissues is the results of animal studies. However, the metabolism, the genome, the physiology and colonic microflora of animals are much different from those of humans. Accordingly, these results are needed to be carefully translated in humans. In addition, a variety of employed animal models does not mimic the progression of the human disease.

The controversial results obtained from human trials might reflect these drawbacks and further be amplified by the human inter-individual variability in the colonic microflora composition that might affect the absorption and the production of the polyphenol active metabolites. In addition, the number of participants in many human studies might be too low and the observation time too short for obtaining an unbeatable result.

Finally, a better knowledge of polyphenols interaction is required for understanding the polyphenols beneficial effects observed in epidemiological studies.

## 8. Conclusions

Polyphenols possess many biological activities, including anti-oxidant, anti-inflammatory, anti-microbial, anti-viral and anti-cancer properties. Several epidemiological studies have shown a relation between a diet rich in polyphenols and the prevention of various human diseases.

Cardiovascular diseases are the mainly cause of mortality and morbidity in the world. Overall, several in vitro and in vivo studies demonstrated the ability of polyphenols to counteract oxidative stress-induced cardiomyocytes damage and death. Polyphenols exert long-lasting effects compared to other direct anti-oxidant agents, because they induce a transcription-mediated signaling, thus activating endogenous anti-oxidant enzymatic defense systems.

In light of the results from in vitro, in vivo and in some clinical trials, and of the activity of polyphenols in the regulation of oxidative stress and inflammation, polyphenols could be useful for the design of novel agents for the treatment of cardiovascular diseases [213]. However, it is necessary a better understanding of the reason why not all individuals show cardiovascular benefits after administration with dietary polyphenols. This knowledge is essential for the universal and definite acceptance of the clinical usefulness of polyphenols in cardiovascular disease.

## Figures and Tables

**Table 1:** In vitro protective effects of polyphenols against the oxidative stress-induced cardiotoxicity.

Polyphenol	Cardiac Damage Inducers	Cell Type	In Vitro Effects	Ref.
Quercetin	DOX H_2_O_2_ A/R Xanthine/XO	H9c2 cells	↓ Apoptosis, ROS, LDH release ↓ DNA fragmentation ↓ Bid, p53 and NADPH oxidase ↓ ERK1/2, Akt, p38, JNK, TNF-α ↓ Phospho-ERK1/2 and –Akt ↑ Phospho-c-Jun and -PKC_Ɛ_ ↓ ∆ψm loss and Ca^2+^ ↓ Phospho-MAPKAPK-2 and caspase 3	[45,48,52,53,57]
3′-*O*-methyl quercetin	H_2_O_2_	H9c2 cells	↓ Apoptosis and LDH release	[45]
Hydroxytyrosol	Xanthine/XO	H9c2 cells	↑ Phopsho-ERK1/2 and -Hsp-27 ↓ Phospho-c-Jun	[57]
Taxifolin	Ang II	Neonatal rat cardiomyocytes	↓ ROS and hypertrophy	[56]
Rhamnetin	H_2_O_2_	H9c2 cells	↓ Apoptosis and ROS ↑ CAT and MnSOD ↓ Phospho-Akt/GSK-3β, -ERK1/2, -p38 and -JNK	[46]
Isorhamnetin	DOX	H9c2 cells	↓ Apoptosis and ROS ↓ LDH release and lipid peroxidation ↑ Anti-oxidant markers ↓ Bax/Bcl-2 ratio, p53, caspases 9 and 3, PARP and cytochrome *c* release ↓ Phospho-ERK, -p38 and -JNK	[49]
Dihydromyricetin	DOX	Primary myocardial H9c2 cells	↓ Apoptosis and ROS ↑ GSH ↓ Nuclear damage, caspase 3 and 8, PARP, ∆ψm loss and Bax/Bcl-2 ratio	[51]
Kaempferol	DOX A/R	H9c2 cells Neonatal primary rat cardiomyocytes	↓ Apoptosis and ROS ↓ LDH release ↑ SIRT1, ∆ψm and Bcl-2 ↓ mPTP opening and DNA fragmentation ↓ Phospho-ERK1/2 ↓ p53, cytochrome *c* release, and caspase 3 and PARP cleavage	[50,54]
3′,4′-dihydroxyflavonol	I/R H_2_O_2_	Cardiomyocytes	↓ Superoxide ↑ Phospho-ERK, -MEK and -Akt	[47,55]
Apigenin	A/R	H9c2 cells	↓ Apoptosis and ROS ↓ LDH release and cytochrome *c* release	[58]
Apigenin glucoside, vitexin	A/R	Neonatal rat cardiomyocytes	↓ Apoptosis and ROS ↓ LDH and CK release ↑ Phospho-ERK1/2	[59]
Baicalein	Ipoxia I/R DOX	Chick cardiomyocytes H9c2 cells hESC-CMs	↓ Apoptosis and ROS ↓ LDH release ↑ Nrf2 pathway and HO-1 ↑ Contractile activity	[61,62,63,64]
EGCG	H_2_O_2_ I/R DOX Bisulfite/sulfite Lead	Neonatal and adult rat cardiomyocytes H9c2 cells Cultures of cardiomyocytes Rat ventricular myocytes	↓ Apoptosis and ROS ↓ Cellular damage and citosolic Ca^2+^ ↓ LDH release and MDA formation ↑ MnSOD, CAT and GSH-Px ↑ HO-1 and caveolin-1 ↓ β-catenin, N-cadherin and Cx43 ↓ p53, p21, caspase 3 and FasR ↓ STAT-1 activation, telomere attrition and TRF_2_ loss	[65,66,67,68,69,70,72,75,76]
EGCG and TF3	H_2_O_2_	Neonatal rat cardiomyocytes	↓ Cellular damage ↑ Akt, ERK1/2 and p38 MAPK	[65]
(−)-epicatechin, avicularin and quercitrin	BSO	H9c2 cells	↓ Apoptosis and LDH release	[77]
Grape seed proanthocyanidin extract	I/R DOX	Chick cardiomyocytes Primary cultures cardiomyocytes	↓ Apoptosis and ROS ↑ NO and GSH-GSSG ratio ↓ ∆ψm loss and DNA fragmentation ↑ Contractile activity	[71,73]
Malvidin	I/R	Rat cardiomyocytes	↑ LV pressure, Akt, eNOS, ERK1/2, and phospho-GSK3β	[78]
Cyanidin-3-*O*-glucoside	I/R	Rat cardiomyocytes	↓ Apoptosis and LDH release	[79]
Blueberry phenol fractions	NE	Adult rat cardiomyocytes	↓ Apoptosis and calpains ↑ SOD and CAT	[80]
Narigenin	H_2_O_2_	H9c2 cells	↓ Apoptosis and lipid peroxidation ↑ GSH-Px, GST, CAT, Nrf2, GCL and NQO-1	[81]
Naringin	High glucose	H9c2 cells	↓ Apoptosis and ROS ↑ GSH-Px, SOD, CAT and Bcl-2 ↓ ∆ψm loss, p53, Bax, Bad, caspase, release of cytochrome c ↓ ERK1/2, p38 MAPK, JNK and leptin	[82,83,84,85]
Naringenin-7-*O*-glucoside	DOX	H9c2 cells	↓ Apoptosis and ROS ↑ SOD, CAT, GSH-Px and NQO-1 ↓ CK, LDH, caspase 9 and 3 mRNA ↑ ERK, Nrf2, HO-1 and Bcl-2	[86,87,88]
Hesperetin	LPS	H9c2 cells	↓ Apoptosis ↑ Bcl-2 ↓ Bax and phopsho-JNK	[89]
Puerarin	Ang II	Neonatal murine cardiomyocytes	↓ ROS ↓ NADPH oxidase ↓ ERK 1/2, JNK and AP-1	[90]
Calycosin	H_2_O_2_	H9c2 cells	↓ Apoptosis ↑ ERα/β and Akt	[91]
Licochalcone D	I/R	Rat cardiomyocytes	↓ Caspase 3 and PARP ↓ IL-6, TNF-α, CRP, LDH, CK, MDA, NO, NF-κB and p38 MAPK ↑ SOD, GSH/GSSG ratio, eNOS and Akt	[92]
Isoliquiritigenin	I/R	Cardiomyocytes	↓ ROS and mitochondrial potential ↑ AMPK and ERK	[94]
Safflor yellow A	A/R	Neonatal rat cardiomyocytes	↓ LDH, CK, MDA and Bax ↑ SOD, CAT, GSH, GSH-Px and Bcl-2	[95]
Hydroxysafflor yellow A	A/R	H9c2 cells	↓ Apoptosis ↑ Nrf2	[96]
Chalcone derivative L6H9	Glucose	H9c2 cells	↓ ROS, Hypertrophy and fibrosis ↓ Bax, caspase 9 and 3 ↓ IL-6, TNF-α, COX and NF-κB ↑ Nrf2, HO-1, NQO-1 and GCLC	[97]
Aspalathin	Hyperglycemia	H9c2 cells	↓ ROS ↓ DNA nick and ∆ψm loss ↑ GSH, SOD and Bcl-2/Bax ratio	[99]
GLG	H_2_O_2_	H9c2 cells	↓ Apoptosis, ROS and hemolysis ↓ Caspase 3 and nuclear condensation and fragmentation	[100]
CSM-bark extract	H_2_O_2_	Neonatal cardiomyocytes	↓ Apoptosis and ROS	[101]
Hispidin	H_2_O_2_	H9c2 cells	↓ Apoptosis, ROS and LDH release ↓ DNA fragmentation, caspase 3 and Bax ↑ HO-1, CAT, Bcl-2, Akt/GSK3β and ERK 1/2	[102]
Methyl gallate	H_2_O_2_	Neonatal rat cardiomyocytes	↓ Apoptosis and ROS ↓ DNA damage, caspase 3 and ∆ψm loss ↑ GSH	[103]
Pyrrolidinyl caffeamide	H_2_O_2_	HL-1 cells	↓ Apoptosis and ROS ↑ CAT, MnSOD, HO-1 and phospho-Akt	[104]
Ferulic acid	High glucose	Cardiomyocytes	↓ Apoptosis and ROS ↑ GSH, Nrf2, HO-1 and Keap-1	[105]
MPE	Malathion	H9c2 cells	↓ ROS, DPPH, ABTS and NO ↑ Integrity of extra cellular matrix components	[106]
Hydroxycinnamic acids	DOX	Neonatal rat cardiomyocytes	↓ Cellular damage and lipid peroxidation	[107]
Danshensu	I/R	H9c2 cells	↓ Apoptosis, ROS, LDH release, CK, MDA and caspase 3 ↑ SOD, CAT, GSH-Px, HO-1, Bcl-2/Bax ratio, PI3K/Akt and ERK1/2	[108,109]
Sch B	I/R	H9c2 cells	↓ Apoptosis, inflammation and ROS ↓ Bax/Bcl-2 ratio, NF-κB ↑ ERK and Nrf2	[110,111]
Syringaresinol	I/R	H9c2 cells	↓ ROS, MDA, Bax/Bcl-2 ratio, caspase 3 and HIF-1 ↑ FoxO3 and anti-oxidant markers	[113]
Sesamin	DOX Ang II	H9c2 cells	↓ Apoptosis, ROS and MDA ↓ Caspase 3, p47phox and ∆ψm loss ↑Bcl-2, SOD and T-AOC	[114,115]
Resveratrol	H_2_O_2_ Hypoxia DOX AZT As_2_O_3_ High glucose Iron	H9c2 cells Neonatal rat ventricular cardiomyocytes Human and rat primary cardiomyocytes	↓ Apoptosis, necrosis, autophagy, mitochondrial dysfunction and cell injury ↓ ROS, NADPH oxidase, LDH release, FoxO1, CaMKII ↓ Bax, phospho-p38 and -JNK ↑ SIRT1, Bcl-2, phospho-Akt and -ERK ↑ SOD, CAT	[116,117,118,119,120,121,122,123,124,125,126,127]
Polydatin	Phenylephrine	Neonatal rat cardiomyocytes	↓ ROS and RhoA/ROCK	[128]
Bakuchiol	I/R	Rat cardiomyocytes	↓ Apoptosis ↑ SIRT1, SDH, cytochrome *c* oxidase and SOD	[129]
Curcumin	TNF-α Peptidoglycan H/R I/R Glucose	Rat cardiomyocytes	↓ Apoptosis, ROS, NADPH oxidase, MDA, lipid peroxidation and protein carbonylation ↓ Bax, cytochrome *c* and cardiolipin release, FoxO1, TLR2 and MCP-1 ↑ Bcl-2, SDH, COX, SOD, SIRT1, Akt and phospho-GSK-3β	[131,132,133,134]
Nanocurcumin	Hypoxia	H9c2 cells HVCM	↓ Apoptosis, hypertrophy and ROS ↓ HIF-1α, caspase 3 and 7, p53 translocation ↓ AMPKα, p-300 HAT, LDH, acetyl-CoA and ∆ψm loss ↑ c-Fos, c-Jun and ATP	[135,136]
Curcumin analogue 14p	I/R	H9c2 cells	↓ Apoptosis, ROS and MDA ↑ Nrf2, SOD	[138]
Salvianolic acid B	TNF-α	HASMC	↓ ROS, NADPH oxidase, MMP-2	[141]
Silymarin	Copper-ascorbate	Neonatal rat cardiomyocytes	↓ ROS, NO, protein carbonylation and lipid peroxidation ↑ mitochondrial function, GSH, GSH-Px, GR, SOD, PDH	[143]
Silibinin	H_2_O_2_ Phenylephrine	H9c2 cells	↓ Apoptosis, DNA damage, ROS ↓ ERK and Akt	[144]
2,3-dehydrosilybin	H/R	Neonatal rat cardiomyocytes	↓ ROS, protein carbonylation and LDH release	[145]
TFCC	DOX	H9c2 cells	↓ Apoptosis, ROS, MDA and LDH release ↓ DNA fragmentation, caspase 3, cytochrome *c* release, Bax/Bcl-2 ratio, p53, phospho-ERK, -p38 and -JNK ↑ SOD, CAT, GSH-Px, phospho-Akt and PI3K	[147]

Abbreviations: ↑: increase; ↓: decrease; DOX: doxorubicin; H_2_O_2_: hydrogen peroxide; A/R: anoxia/reoxygenation; XO: xanthine oxidase; ROS: reactive oxygen species; ERK: extracellular signal-regulated kinase; JNK: c-jun N-terminal kinase; TNF-α: tumor necrosis factor-α; PKC_Ɛ_: Protein kinase C epsilon type; ∆ψm: mitochondrial membrane potential; LDH: lactate dehydrogenase; MAPKAPK-2: mitogen-activated protein kinase-activated protein kinase 2; Hsp: heat shock protein; Ang II: angiotensin II; CAT: catalase; MnSOD: manganese superoxide dismutase; GSH: glutathione; SIRT1: sirtuin 1; mPTP: mitochondrial permeability transition pore; I/R: ischemia/reperfusion; CK: creatin kinase; H/R: hypoxia/reoxygenation; hESC-CMs: human embryonic stem cells-derived cardiomyocytes; Nrf2: nuclear factor erythroid 2-related factor 2; HO-1: heme oxygenase-1; EGCG: (−)-epigallocatechin-3-gallate; MDA: malondialdehyde; GSH-Px: glutathione peroxidase; TF3: theaflavin-3,3′ digallate; BSO: buthionine-(*S*,*R*)-sulfoximine; NO: nitric oxide; GSSG: glutathione disulfide; LV: left ventricular; NE: norepinephrine; GCL: glutamate cysteine ligase; NQO-1: NAD(P)H:quinone oxidoreductase 1; LPS: lipopolysaccharide; ER: estrogen receptor; IL: interleukin; CRP: C reactive protein; eNOS: endothelial nitric oxide synthase; COX: cycloxygenase; GCLC: glutamate cysteine ligase catalytic subunit; GLG: Gui-ling-gao; CSM: *Castanea Sativa* Mill.; MPE: gallic acid-enriched methanolic *Syzygium cumini* pulp extract; DPPH: 1,1-diphenyl-1-picrylhydrazyl; ABTS: 2,2′-azino-bis (3-ethylbenzthiazoline-6-sulfonic acid); Sch: schisandrin; HIF-1: hypoxia inducible factor-1; FoxO: Forkhead box protein O; T-AOC: total antioxidant capacity; AZT: azidothymidine; As_2_O_3_: arsenic trioxide; CaMKII: Ca^2+^/Calmodulin-dependent protein kinases II; GR: glutathione reductase; GST: glutathione s-transferase; TLR2: Toll-like receptor 2; MCP-1: monocyte chemoattractant protein; SDH: succinate dehydrogenase; HVCM: primary human ventricular cardiomyocytes; HASMC: human aortic smooth muscle cells; MMP: metalloproteinase; PDH: piruvate dehydrogenase; TFCC: Clinopodium chinense (Benth.) O. Ktze.

**Table 2:** In vivo protective effects of polyphenols against the oxidative stress-induced cardiotoxicity.

Polyphenol	In Vivo Model	Protective Effects	Ref.
Quercetin	Mice treated with DOX	↑ Cardiac function ↓ ROS and lipid peroxidation ↑ Bmi-1 and SOD expression	[48]
	Rats treated with DOX	↓ Blood pressure and heart rate increase ↓ Cellular damage ↓ MMP-2 activation and apoptosis ↑ SOD activity	[156]
Vincristine and quercetin	Rats exposed to isoproterenol	↓ CK-MB, LDH, ALT, cTnT ↓ Lipid peroxidation ↑ SOD, CAT, GR, GSH-Px activities ↓ Heart rate and ST-segment elevation	[161]
Taxifolin	Mouse model of TAC	↓ Pressure overload, fibrosis, ROS, MDA, HNE ↓ Cardiac remodeling and ventricular dysfunction ↓ ANP, BNP, β-MHC expression ↓ Phospho-ERK1/2, phospho-JNK1/2, Smad2	[56]
DiOHF	Sheep model of I/R injury	↓ ROS, neutrophil accumulation, LVDP, infarct size ↑ Myocardial function	[55]
Isorhamnetin	Rats treated with DOX	↓ Cardiac enzymes, apoptosis, ROS, lipid peroxidation ↑ Anti-oxidant enzymes	[49]
Rutin	Rats exposed to sodium fluoride	↓ Cardiac dysfunction, cardiac serum markers ↓ Lipid peroxidation and DNA fragmentation ↑ SOD, CAT, GSH levels	[160]
Dihydromyricetin	Mice treated with DOX	↑ Survival rate ↓ AST, CK-MB, LDH activities	[51]
Kaempferol	Rats treated with DOX	↑ Body and heart weights, SOD, CAT ↓ LDH levels, apoptosis, mitochondrial damage	[50]
	Rat model of I/R injury	↑ Cardiac function, SOD activity, GSH/GSSG ratio ↓ CK, LDH, MDA levels, infarct size, apoptosis	[157]
Astragalin	Rat model of I/R injury	↑ Cardiac function, SOD activity, GSH/GSSG ratio ↓ CK, LDH, MDA levels, infarct size, apoptosis	[158]
Baicalein	Murine model of I/R injury	↓ Infarct size, apoptosis, pro-inflammatory cytokines ↓ ROS, MDA levels ↑ GSH-Px	[162]
Apigenin	Rat model of I/R injury	↓ Infarct size, apoptosis, CK, LDH, MDA levels ↑ SOD	[163]
Vitexin	Rat model of I/R injury	↑ Cardiac function, SOD activity ↓ Infarct size, apoptosis, inflammatory cytokines ↓ CK, LDH, MDA	[164]
Luteolin	Rat model of I/R injury	↑ Cardiac function, MnSOD activity ↓ LDH, MDA levels	[165]
Breviscapine	Rat model of I/R injury	↓ ICAM-1, ROS, MDA ↑ SOD, GSH-Px activities	[166]
Green Tea Exctract (GTE)	Rat model of I/R injury	↓ Infarct size, apoptosis ↑ GSH, GCL, QR	[167]
	Rats treated with DOX	↓ AST, CK, LDH, lipid peroxidation ↑ Cyt P450, GSH, GSH-Px, GR, GST, SOD, CAT	[169]
EGCG, quercetin	Rats with cardiac hypertrophy	↓ Systolic blood pressure, heart weight indices, MDA ↑ SOD, GSH-Px activities, apoptosis	[67,171]
GSP	Rats treated with cadmium	↓ Cardiac damage, CK-MB, AST, ALT, ALP, LDH ↓ Pro-inflammatory cytokines, apoptosis ↑ GSH-Px, GR, GST, SOD, CAT, G6PD	[172]
Procyanidins	Rats treated with DOX	↑ Cardiac function ↓ Lipid peroxidation	[170]
BAE	Rats treated with CTX	↑ Cardiac function , IL-10, SOD, GSH ↓ Apoptosis, pro-inflammatory cytokines, MDA	[174]
Hesperidin	Rat model of I/R injury	↑ Cardiac function ↓ Apoptosis, oxidative stress	[176]
Naringin	Rat model of I/R injury	↓ CK-MB, LDH, apoptosis, infarct size, inflammation ↑ SOD, GSH-Px	[177]
Hesperetin	Rats treated with DOX	↓ MDA, DNA damage ↑ GSH	[178]
Hesperidin	Rats treated with isoproterenol	↓ Lipid peroxidation ↓ CK, CK-MB, LDH, AST, ALT, cTnI, cTnT ↑ SOD, CAT, GSH-Px, GST, GR	[180]
Hesperidin, naringin	HFD/STZ-induced diabetic rats	Prevention of diabetic complications ↓ MDA, NO ↑ SOD, CAT, GSH-Px, GR	[182]
Puerarin	Mice treated with Ang II	↓ Cardiac hypertrophy, HW/BW, LVW/BW	[90]
	Rats subjected to severe burn	↓ CK-MB, cTnT, MDA, MPO	[184]
Calycosin-7-*O*-β- d -glucoside	Rat model of I/R injury	↑ Cardiac function, SOD activity ↓ Infarct size, CK, LDH, MDA, apoptosis	[183]
Chalcone derivative L6H9	STZ-induced diabetic mice	↓ Cardiac damage and fibrosis ↓ ROS, TNF-α, IL-6, COX2, Bax ↑ HO-1, NQO-1, GCLC, Bcl-2	[97]
Licochalcone B	Rat model of I/R injury	↓ Apoptosis, MDA, LDH, CK, TNF-α ↑ LVDP, SOD, GSH/GSSG ratio	[185]
Cl-chalcone, F-chalcone	Rat model of I/R injury	↓ Infarct size, lipid peroxidation, MDA ↑ SOD, CAT	[186]
Pyrrolidinyl caffeamide (PLCA)	Rat model of I/R injury	↓ Troponin, MDA, MPO ↑ Cardiac function, CAT, HO-1, MnSOD	[104]
Danshensu	I/R in spontaneously hypertensive rats (SHR)	↓ Blood pressure increase, arrhythmias, HW/BW ↑ NO content, iNOS activity	[188]
	Rat model of I/R injury	↓ Infarct size, CK-MB, cTnI	[108]
Shenge	Rats subjected to LAD	↓ ST-segment elevation, infarct size ↓ CK-MB, LDH, MDA ↑ SOD activity	[189]
Schisandrin B (Sch B)	Rats treated with DOX	↓ CK, CK-MB, LDH, AST, MDA, MMP ↓ Cardiac damage, cell death ↑ GSH, GSH/GSSG, GR, GST, GSH-Px, SOD	[190]
	Mice treated with DOX	↓ Cardiac damage, apoptosis, DNA damage ↓ ROS, MDA, TNF-α, IL-1β, IL-6, MMP-2, MMP-9 ↑ GSH, LV performance	[191]
	Mouse model of myocardial infarction (MI)	↑ Survival rate, heart function, eNOS ↓ Infarct size, TGF-β1, TNF-α, IL-1β, Bax/Bcl-2	[111]
	Rat model of I/R injury	↑ GSH ↓ LDH	[110]
Magnolol	Rat model of I/R injury	↓ Infarct size, apoptosis, myocardial dysfunction	[193]
Sesamin	SHR rats	↓ Cardiac fibrosis, systolic blood pressure ↓ HW/BW, LVW/BW, MDA, TGF-β1 ↑ Cardiac total anti-oxidant capability	[192]
	Rats treated with DOX	Normalization QT intervals, QRS complexes ↑ SIRT1 activation, MnSOD	[114]
Deoxyshizandrin (DSD) + Schisantherin (STA)	Rat model of I/R injury	↓ Infarct size, LVDP, arrhythmias, MDA ↑ LVSP, SOD	[112]
Resveratrol	SHR rats	↓ H_2_O_2,_ left ventricular hypertrophy ↑ CAT activity	[119,194]
	Mice treated with arsenic trioxide (As_2_O_3_)	↓ QT-interval prolongation, cardiac damage, LDH ↑ GSH-Px, CAT, SOD	[125]
	Mice treated with LPS	↑ SERCA2a, Nrf2 ↓ MDA, HNE	[199]
	Rats treated with cisplatin	↓ LDH, CK, MDA ↑ SOD, GSH, GSH-Px, CAT	[200]
	Rats treated with DOX	↓ Cardiac dysfunction, apoptosis, MDA, CK, LDH ↑ SIRT1, GSH	[196,197]
	STZ-induced diabetic mice	↓ Apoptosis, p62 ↑ Cardiac function, SIRT1, autophagy	[195]
Polydatin + vitamin C	Rats treated with DOX	↓ ROS, MDA, CRP, ST and QT intervals ↑ GSH-Px, SOD	[198]
Polydatin	Mice subjected to TAC	↓ Cardiac hypertrophy	[128]
Curcumin	Rat model of I/R	↓ Lipid peroxidation ↑ Cardiac function, SOD, CAT, GSH, GSH-Px	[201]
	Rats treated with isoprenaline	↓ Apoptosis, MPO, MDA ↑ CAT, GSH	[202]
	Rat model of I/R	↑ SIRT1, Bcl-2, SDH, COX ↓ Bax, CK, LDH	[132]
	STZ-induced diabetic rats	↓ MDA, hypertrophy, fibrosis, ventricular dysfunction ↑ GSH-Px	[203]
Curcumin + nebivolol	Rats treated with DOX	↑ Survival rate, SOD, GSH-Px, Body and heart weights ↓ Cardiac damage, lipid peroxidation, NO ↓ QT and ST intervals	[204]
Oleuropein	Rats treated with DOX	↓ CK, CK-MB, LDH, ALT, AST, apoptosis ↓ MDA, protein carbonyl, nitrotyrosine, iNOS	[205,206]
Silymarin	Rats treated with DOX	↓ CK, LDH, creatinine, urea, MDA ↑ GSH	[207]
	Rats treated with cisplatin	↓ LDH, CK, CK-MB, cTnI, MDA ↑ GSH, SOD	[209]
	Mice treated with acrolein	↓ Lipid peroxidation, apoptosis, MDA, cTnI, CK-MB ↑ GSH, SOD, CAT	[210]
Silibinin	Rats treated with arsenic	↑ Cardiac function, Nrf-2, HO-1 ↑ SOD, CAT, GSH-Px, GST, GR, G6PD ↓ CK-MB, LDH, AST, ALT, ALP, HW/BW	[211]
*Clinopodium chinense* (Benth.) O. Ktze (TFCC)	Rats treated with DOX	↑ Body and heart weights ↓ CK, AST, LDH, MDA, apoptosis ↑ SOD, CAT, GSH-Px	[147]

Abbreviations: ↑: increase, ↓: decrease; DOX: doxorubicin; ROS: reactive oxygen species; SOD: superoxide dismutase; MMP: metalloproteinase; CK-MB: creatin kinase-MB; LDH: lactate dehydrogenase; ALT: alanine aminotransferase; cTn: cardiac troponin; CAT: catalase; GR: glutathione reductase; GSH-Px: glutathione peroxidase; TAC: transverse aortic constriction; MDA: malondialdehyde; HNE: 4-hydroxy-2-nonenal; ANP: atrial natriuretic peptide; BNP: brain natriuretic peptide; ERK: extracellular signal-regulated kinase; JNK: c-jun *N*-terminal kinase; DiOHF: 3′,4′-dihydroxyflavonol; I/R: ischemia/reperfusion; LVDP: left ventricular end diastolic pressure; GSH: glutathione; AST: aspartate aminotransferase; GSSG: glutathione disulfide; MnSOD: manganese superoxide dismutase; EGCG: (−)-epigallocatechin-3-gallate; IL: interleukin; GCL: glutamate cysteine ligase; QR: quinone reductase; Cyt: cytochrome; GST: glutathione s-transferase; GSP: grape seed proanthocyanidins; ALP: alkaline phosphatase; G6PD: glucose-6-phosphate dehydrogenase; BAE: blueberry anthocyanins-enriched extracts: CTX: cyclophosphamide; HFD/STZ: high fat diet/streptozotocin; Ang II: angiotensin II; HW/BW: heart weight/body weight ratio; LVW/BW: left ventricular weight/body weight ratio; MPO: myeloperoxidase; TNF-α: tumor necrosis factor-α; COX: cyclooxygenase; HO-1: heme oxygenase-1; NQO-1: NAD(P)H:quinone oxidoreductase 1; GCLC: glutamate cysteine ligase catalytic subunit; NO: nitric oxide; iNOS: inducible nitric oxide sinthase; LAD: left anterior descending; SHR: spontaneously hypertensive rats; SIRT1: sirtuin 1; LVSP: left ventricular systolic pressure; SERCA: sarcoplasmic reticulum Ca^2+^ ATPase; Nrf2: nuclear factor erythroid 2-related factor 2; CRP: C reactive protein; SDH: succinate dehydrogenase.
